# Qualitative evaluation of the barriers and facilitators to a retrospective hepatitis C virus patient re-engagement exercise in England

**DOI:** 10.1136/bmjopen-2025-104546

**Published:** 2025-11-13

**Authors:** Avelie Stuart, Carina Hörst, David Etoori, Fabiana Lorencatto, William Rosenberg, Catherine Lowndes, Ruth Simmons, Sema Mandal, Mark Gillyon-Powell, Monica Desai

**Affiliations:** 1 UK Health Security Agency, London, UK; 2 Centre for Clinical Research, Epidemiology, Modelling and Evaluation (CREME), Institute for Global Health, University College London, London, UK; 3 NIHR Health Protection Research Unit in Blood Borne and Sexually Transmitted Infections, London, UK; 4 Centre for Behaviour Change, University College London, London, UK; 5 Institute for Liver and Digestive Health, Division of Medicine, University College London, London, UK; 6 Specialised Commissioning, NHS England, London, UK

**Keywords:** Public health, Patient Care Management, Hepatology, QUALITATIVE RESEARCH

## Abstract

**Abstract:**

**Objectives:**

The UK Health Security Agency and the National Health Service England (NHSE) led a hepatitis C virus (HCV) patient re-engagement exercise beginning in 2018, which entailed sharing public health surveillance data with NHSE Operational Delivery Networks (ODNs) in England. The ODNs used the data to contact and offer testing and treatment to people historically diagnosed with HCV, but who did not have evidence of successfully clearing the virus. A quantitative evaluation found that of 55 329 individuals whose details were shared with ODNs, around 13% had treatment after the exercise commenced. This qualitative evaluation aims to identify the barriers and facilitators to the re-engagement exercise as reported by ODN staff.

**Design:**

Semistructured interviews. The topic guide and analysis were guided by the Theoretical Domains Framework, using a combined deductive framework and inductive thematic analysis approach.

**Setting and participants:**

21 staff from 13 ODNs. The sampling frame was designed to capture participants from all regions of England and with varied outcomes from the re-engagement exercise.

**Results:**

Interviewees reported the most barriers in environmental context and resources (including staffing limitations, interruptions during COVID-19, restricted laboratory access), and social influences (with limited responses from general practitioners and patients). Interviewees discussed whether it was appropriate for ODNs and individual staff to be assigned the data validation work and reported some stress and memory/attention barriers due to the volume of the exercise. They had varied beliefs about the consequences of the exercise, with most believing it was worthwhile due to treatment yield, lessons learnt and confirmation that some people had cleared the virus. Further facilitators included the ODN goals fitting with the exercise, and regional resources such as patient databases. Interviewees also reported adaptations to the exercise that facilitated patient contact, and their ongoing work to re-engage patients emphasised outreach partnerships and peer support.

**Conclusions:**

The evaluation revealed insights into methods for re-engaging patients and of sharing and using public health data to support clinical practice. Government support and funding provision for regionally tailored holistic re-engagement approaches, alongside enhancements to health surveillance data, could enable barriers to re-engagement to be overcome.

**STRENGTHS AND LIMITATIONS OF THIS STUDY**

- The sampling frame enabled representation of participants across regions and with different outcomes, and who differed in their return of outcome data; however, not all regional networks are represented.
- The use of the Theoretical Domains Framework enabled a comprehensive analysis of individual, sociocultural and environmental barriers and facilitators.
- Participants had recall issues due to the amount of time since beginning the exercise, which may have biased the results.

## Introduction

 The number of people living with chronic hepatitis C virus (HCV) in England has fallen 30% since 2015 to an estimated 89 900 people in 2020.^1^ An estimated 55 000 of those people had been lost to follow-up at the time.^2^ The re-engagement of these patients is a necessary component of meeting the WHO Global Health Sector Strategy for the elimination of viral hepatitis by 2030.^3^ Following the introduction of highly effective and well-tolerated Direct Acting Antivirals for HCV, a national retrospective patient re-engagement exercise was launched in September 2018 by the UK Health Security Agency (UKHSA) and National Health Service England (NHSE)^2^ to help support people who were diagnosed with HCV historically, but who have no known treatment outcome or clearance of HCV at the time.

Hepatitis C Operational Delivery Networks (ODNs) are secondary care providers that were established by NHSE in 2015 and charged with managing HCV treatment, providing impartial clinical advice and expertise, and coordinating patient pathways across different services and providers, within a geographical catchment area, to ensure equitable and high standards of care. People can be referred to ODNs via primary care, drug and alcohol services and sexual health/genitourinary clinics. The UKHSA is an arms-length government body that conducts hepatitis C surveillance using laboratory records, to support public health responses to infectious diseases. The re-engagement exercise involved UKHSA sharing surveillance data with the 22 ODNs, using individual Data Sharing Agreements with each ODN and approval to share data under Regulation 3 of The Health Service (Control of Patient Information) Regulations 2002.

**Figure 1:**
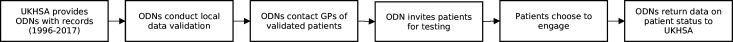
Simplified process diagram of the re-engagement exercise.ODNs may not have carried out these stages in the way depicted. GPs, general practitioners; ODNs, Operational Delivery Networks; UKHSA, UK Health Security Agency.

The UKHSA surveillance data contained lists of individuals with a laboratory record of HCV antibody (indicating they may have HCV and require further investigation to confirm or refute active infection) or PCR RNA (indicating they have active HCV) positive tests from January 1996 to December 2017. Exercise guidance for the ODNs^4^ contained a recommended procedure (see figure 1 for simplified overview) and template letters for general practitioners (GPs) and patients, which were co-produced with the patient advocacy group the Hepatitis C Trust. The General Practice Committee advised that GPs would not have capacity to further support the exercise, thus ODNs were instructed to proceed without a GP response, after giving GPs sufficient time to respond. ODNs were asked to document the exercise and return their process and outcome data to UKHSA.

The re-engagement exercise was publicised in the WHO European Region compendium of good practice in 2020,^5^ and two evaluations have been conducted.^6 7^ The preimplementation qualitative evaluation with ODNs in 2019 found that the data validation (ie, checking patient records against local databases/labs) was time and resource intensive.^7^ The evaluation recommended that ODNs share successful local approaches to improving uptake of testing and treatment. In a phase 2 quantitative evaluation using data from 2022 (when half of the ODNs had returned data),^6^ there were reported gains in re-engagement of confirmed HCV-positive patients (7442–18%—of eligible patients were treated) as well as in determination of another status for individuals, such as a PCR RNA negative result (6435, 11.6%) or that the patient had deceased (2990, 5.4%). However, there remained 33 349 (59%) individuals with unknown HCV status.

ODNs varied in their approaches and faced different circumstances that influenced their treatment yield.^7^ Two ODNs published short reports,^8 9^ with one^8^ stating that they had focused on re-engaging patients for whom they could locate a local positive HCV PCR laboratory test, but that they experienced significant interruption during the COVID-19 pandemic, resulting in few people being treated. This evaluation will further explore the barriers and facilitators faced by ODNs.

This exercise was a first for England, with a sparsity of similar re-engagement research to draw on when designing the exercise. Some HCV and HIV re-engagement investigations have been conceived or conducted since.^10^,^14^ For example, a retrieval exercise was piloted in the Utrecht region in the Netherlands, using HCV laboratory results from 2001 to 2015^12^; patients who were lost to follow-up were invited for free medical check-ups. They reported that 43% of people responded to letters, and there were higher response rates from local residents (61%), compared with those who lived outside of the province (28%). When rolled out nationally, the exercise achieved re-engagement of 14% of the eligible population,^11^ a similar yield to this re-engagement exercise. A similar re-engagement exercise was conducted in Wales, UK, and they reported that 21% of patients who received letters contacted services, and of those, 44% completed treatment.^14 15^ Qualitative evaluations of any such re-engagement exercises are lacking in the published literature.

The delivery and uptake of the re-engagement exercise are forms of human behaviour. Therefore, understanding what helped or hindered this could be facilitated through the application of behavioural science frameworks. One framework widely used to investigate implementation challenges and conduct qualitative evaluations in healthcare is the Theoretical Domains Framework (TDF), which integrates constructs from 33 behaviour change theories into 14 domains representing a broad range of individual, sociocultural and environmental barriers and facilitators to implementation and behaviour change,^16^ including in the context of HCV.^17^,^19^ This study, therefore, applies the TDF to analyse the barriers and facilitators to the re-engagement exercise, as reported by ODN staff who were tasked with validating laboratory records, contacting, testing, treating (if PCR-positive) or otherwise determining the status of the patients from the surveillance data. Due to the amount of time that had passed, we were unable to involve patients in the evaluation. This evaluation will help to build on the evidence of good practice and contribute suggestions for the optimisation of similar re-engagement exercises.

## Methods

### Design

Semistructured interviews were chosen to allow for in-depth exploration of barriers and facilitators, while allowing for some flexibility to expand on interviewees’ responses. We adhered to the Standards for Reporting Qualitative Research in designing the study and writing the report.^20^

### Materials

The interview topic guide (online supplemental Appendix 1) was developed using the TDF.^16^ The topic guide had three broad sections. Section 1 aimed to explore how each ODN conducted the re-engagement exercise. Section 2 aimed to explore barriers and facilitators. Section 3 explored other approaches to re-engagement that the ODN was using and their suggestions for further re-engagement work. The section on barriers and facilitators was semi-structured around the domains of the TDF, with at least one question per domain and flexible follow-on prompts. Table 1 lists each domain from the TDF with an example question from the topic guide. The topic guide was piloted with two hepatology consultants and refined to enhance clarity and flow.

**Table 1:** Example questions from the topic guide for each domain from the TDF

TDF domain	Example question
Behavioural regulation Anything aimed at managing or changing objectively measured actions	What strategies or ways of working within your ODN do you have in place that helped you before you started the exercise?
Beliefs about capabilities Acceptance of the truth, reality or validity about an ability, talent or facility that a person can put to constructive use	How confident did you feel in your ability to conduct the re-engagement exercise?
Beliefs about consequences Acceptance of the truth, reality or validity about outcomes of the exercise	Were there any benefits of conducting the exercise? What about any downsides?
Emotion Complex reactions by which the individual attempts to deal with the feelings invoked by the exercise	Did you have any worries or concerns about the re-engagement exercise?
Environmental context and resources Any circumstance of a person’s situation or environment that discouraged or encouraged them to work on the exercise	To what extent did you feel that your ODN had sufficient resources? (time, staff, information, other resources)
Goals Mental representations of outcomes that individuals want to achieve that relate to the exercise	Did your ODN have any targets related to the re-engagement exercise?
Intention A conscious decision to perform a behaviour or a resolve to act in a certain way	To what extent do you feel it was necessary to conduct the re-engagement exercise?
Knowledge An awareness of the existence of the components of the exercise/how to do the exercise	Did your ODN receive any guidance for conducting the re-engagement exercise?
Memory, attention and decision-making processes The ability to retain information, focus selectively on aspects of the exercise and choose between two or more alternatives	How much of your attention did the re-engagement exercise require?
Optimism The confidence that things will happen for the best or that desired goals will be attained	Were you optimistic or pessimistic that conducting the re-engagement exercise would help re-engage patients in care? Why?
Reinforcement Increasing the probability of a response with a dependent relationship/contingency, between the incentives/benefits/rewards/punishments and the exercise	Were there any incentives in place for your ODN to conduct the re-engagement exercise?
Skills An ability or proficiency in conducting the exercise	In your opinion, did your ODN have sufficient training to carry out the exercise?
Social influences Interpersonal processes that can cause individuals to change their thoughts, feelings or behaviours during their conduct of the exercise	How did patients react to the re-engagement exercise?
Social/professional role and identity Behaviours or qualities of the individual or organisation in their work setting, and how these influenced the exercise	To what extent did you feel it was part of your ODNs responsibility to conduct the re-engagement exercise? Who else was/should have been responsible?

ODN, Operational Delivery Network; TDF, Theoretical Domains Framework.

### Sample and procedures

All 22 ODNs undertook the initial re-engagement exercise. Using the quantitative evaluation data,^6^ we developed a sampling frame so as to interview ODNs from the top, middle and lower third of treatment yields from the re-engagement exercise, and whether or not they returned data to UKHSA. We also interviewed ODN staff across the regions of England: North, South, London, the Midlands and the East of England. We aimed to recruit 15–20 participants, based on guidance for sample sizes in qualitative research.^21^

In November 2023, a study invitation was circulated via senior ODN clinical leads and an ODN newsletter. ODNs selected using the sampling frame were also emailed directly. Additional emails were circulated through contacts of the NIHR Health Protection Research Unit for Blood-Borne and Sexually Transmitted Infections, and UKHSA’s Blood Borne Virus regional facilitators. The inclusion criterion was that interviewees worked for the ODN during the re-engagement exercise.

The interviews were conducted from November 2023 to March 2024, and recorded on Microsoft Teams, and ranged in length from 33 min to 1 hour 5 min (average 47 min). Transcripts were anonymised to ensure confidentiality. The interviewers are trained and experienced in research interviewing and were not known to the ODNs, nor involved in the re-engagement exercise. Rather than data saturation, our decision to end data collection occurred when our recruitment strategy was exhausted, as some staff working on the exercise were no longer employed in the ODN and we had exceeded our estimated sample size. We also observed a high frequency of common themes across the interviews (see Results).

### Data analysis

Transcripts were analysed using NVivo V.14, in line with published guidance on applying the TDF to qualitative data.^16^ This involved a combined deductive framework and inductive thematic analysis approach, following five steps: (1) familiarisation with transcripts, (2) development of codebook using three transcripts coded by the first author to aid the deductive analysis to the TDF domains, (3) discussing and revising the codebook with the team (see online supplemental Appendix 2 for final version), (4) inductively identifying themes within domains using all of the transcripts and (5) writing the report and providing illustrative quotations of the final themes.

The themes are presented in the results section in approximate chronological order according to the stages of the exercise (figure 1), although some themes were relevant throughout the exercise. Here, we also note any observed patterns between either: (1) barriers/facilitators and whether the interviewee’s ODN returned re-engagement data to UKHSA or not or (2) barriers/facilitators and the interviewee’s ODN treatment yield, derived from the quantitative evaluation.^6^ While not a quantitative analysis, such patterns may indicate more substantial barriers/facilitators. To retain anonymity, we do not link findings to specific ODNs. Due to the flexible conduct of the exercise reported by interviewees, we also identified themes about the adaptations that ODNs made to their study protocols due to barriers that they encountered, which became facilitators in themselves. Finally, we summarise interviewees’ recommendations and reflections on other successful re-engagement work they have conducted.

### Patient and public involvement

This study did not employ patient or public involvement; however, it is part of a series of studies, including on patient experiences of being re-engaged in care by the ODNs.

## Results

### Participants

Table 2 details the numbers of participants per ODN and region of England. Each ODN has a different structure and assigned the exercise to different staff, therefore, interviewees’ professional roles included consultants (n=4), clinical staff (n=2), managers/administrators (n=11), ODN leadership team members (n=2) and patient coordinators (n=2). Due to the small sample pool and risk of deanonymisation, we do not specify further information about interviewees.

**Table 2:** Number of interview participants and their characteristics

Participant characteristics	Number
Number of participants	21
Number of ODNs with which participants were affiliated	13
Number of participants from ODNs that did not return re-engagement exercise data	13
Participants per region of England London Midlands and East England North England South England	4 4 9 4

ODNs, Operational Delivery Networks.

### Barriers and facilitators

Table 3 contains a summary of the main themes, organised approximately by the stage in the exercise where they primarily or first arose. We followed guidance on the TDF^16^ on how to select the main themes—using a combination of theme frequency, themes that participants emphasised as important, and themes involving learnings for any similar re-engagement exercises. The remaining themes can be found in online supplemental Appendix 3.

**Table 3:** Summary of themes by theoretical domain—mixed themes acted as both barriers and facilitators

Theoretical domain—theme name	Number of participants	Barrier/mixed/ facilitator
Goals		
The exercise fitted well with targets and/or elimination goals	9	Facilitator
Environmental resources and context		
Low staff and staff turnover caused disruption	16	Barrier
Referral processes, data access and governance issues	7	Barrier
*Laboratories*: Some existing access, but no capacity to gain access to all laboratories	14	Mixed
COVID-19 affected the exercise	15	Mixed
There were useful ODN/Trust resources, databases and systems	10	Facilitator
Social/professional role and identity		
Appropriateness of ODN conducting the re-engagement exercise	17	Mixed
Appropriateness of individual roles for tasks	16	Mixed
Skills		
IT/data/screening skills training were needed but not provided	8	Barrier
Knowledge		
There was a lack of up-to-date data about patients	11	Barrier
Emotion		
Stress and other negative emotions	12	Barrier
Memory, attention and decision-making processes		
The exercise required a lot of time and attention	16	Barrier
Social influences: UKHSA/NHSE/ODN/GPs		
Perceived pressure to conduct the exercise	10	Mixed
Interactions with UKHSA/NHSE during the exercise	17	Mixed
ODN was collaborative	10	Facilitator
Cooperation/support, or lack thereof, from GPs	19	Mixed
Beliefs about consequences		
Worried about people’s responses to the exercise before starting	8	Mixed
Social Influences: People/patients		
It was difficult to make contact with people/patients	12	Barrier
Most people/patients happy to be contacted, but a small number of difficult conversations were had	15	Mixed
Beliefs about consequences		
Treatment yield and other outcomes from the exercise	19	Mixed

GPs, general practitioners; NHSE, National Health Service England; ODNs, Operational Delivery Networks; UKHSA, UK Health Security Agency.

### Goals

#### The exercise fitted well with targets and/or elimination goals (facilitator)

The goals theme was relevant at all stages of the exercise, but particularly at the beginning, as it influenced the ODNs’ ability to resource and prioritise the exercise. When interviewees reported that their ODN had goals that fitted well with the re-engagement exercise, the exercise enabled them to achieve these goals and to prioritise the work.

[the re-engagement exercise] was one of the key things that we were doing ‘cause, I mean my view was well, we know that there’s several probably thousand people that we know have got Hep C vs we can go and test. And it just seemed an obvious place to go. So it was a priority for us. (Participant 23)

Related ODN goals that helped them prioritise the exercise included HCV elimination goals, National Health Service (NHS) Commissioning for Quality and Innovation targets (used as part of calculating the annual payment for NHS Trusts and Foundation Trusts under the NHS Payment Scheme), and goals to complete the re-engagement exercise before other projects started. Interviewees in this theme were from ODNs with the highest third of treatment yield.

### Environmental context and resources

#### Referrals, data access and information governance issues (barrier)

Initiating the exercise often began with internal governance reviews and setting up new patient processes, as the patients had not been referred to the ODN (eg, from primary care). Additionally, some staff had to set up honorary contracts to access other hospitals’ systems within the catchment area of the ODN, with varied capacity to do so.

I didn’t actually work out of [the other] hospitals, but I contacted them from [my hospital] so it was quite a large thing to set up because you know …. setting up an honorary contract actually had to go and do courses in order to use their systems. (Participant 11)

The governance boards at each Hospital Trust often reviewed the exercise protocol, resulting in disparate delays across multiple hospitals within an ODN. Some interviewees reported being unable to gain access or approvals at all sites.

#### Laboratories: some existing access, but no capacity to gain access to all laboratories (mixed)

The next process required in the exercise was to validate the surveillance data with local laboratories. Where the ODN had good links with their laboratories prior to the exercise, laboratory access acted as a facilitator.

I mean, we already had good links with our labs because we were setting up alerts, so we could kind of identify and get reports from them and that kind of was quite helpful as part of the data cleansing initially (Participant 1)

However, some interviewees said they did not have capacity to gain access to smaller private regional laboratories. If they did not have access, they were unable to determine if patients had already had a PCR RNA test unless the surveillance data contained the necessary information (often, only the antibody result was available). Interviewees from ODNs who did not return data to UKHSA more commonly reported lack of access to laboratory data.

#### Low staff and staff turnover caused disruption (barrier)

Most interviewees reported challenges in adequate staffing of the re-engagement exercise, with existing staff not having extra capacity and lack of resources to hire more staff.

as was probably the case with everyone, there wasn't a dedicated team just for that programme. And so, it was sort of done at the end of… well it was done alongside lots of other things (Participant 25)

The exercise was said to depend on one or two key staff, with small numbers of nurses supporting. When key staff left their role, there was difficulty replacing them and continuity issues were reported.

#### COVID-19 affected the exercise (mixed)

For many of the interviewees who worked on the exercise from 2020 onwards, substantial disruption was caused by the COVID-19 pandemic and the associated social restrictions and staff redeployment.

We still carried on treating and testing patients for hepatitis C, but this exercise really went on hold and we came back to it after COVID essentially. (Participant 12)

Interviewees from ODNs with the lowest third of patients treated since the re-engagement exercise reported this issue more frequently. Unexpectedly, a few interviewees said the pandemic freed up time that allowed them to focus on the exercise.

#### There were useful ODN and Trust resources, databases and systems (facilitator)

Some ODNs reported benefiting from existing resources such as their own treatment databases and electronic patient record systems.

…we had quite good systems in place for managing Hep C treatment and understanding who had already gone through that (Participant 1)

Additionally, ODN-external hospital teams (eg, data teams, patient liaison services) with capacity to take on work alleviated some of the burden of the data validation phase. Hospital patient advice and liaison departments, and phone lines managed by the clinical nurses, were able to support patients after letters were sent out. Some ODNs had similar pre-existing template letters or protocols that they felt helped them. Finally, where ODNs were able to hire staff at the same time as the exercise, then the work was integrated into those roles. Interviewees reporting these resources were commonly from ODNs who had the highest treatment yield.

### Social/professional role and identity

#### Appropriateness of ODNs conducting the re-engagement exercise (mixed)

Related to environmental context and resources, it was also during the early stages that some interviewees reported doubts about whether the data should have been shared with the ODN without further data linkage or cleaning. They questioned whether antibody test results could have instead been assigned to the original test requester (eg, GPs, specific hospitals) to follow-up. One interviewee said the ODNs were not trained in public health screening or surveillance, in reference to the need for confirmatory testing where only antibody test results were available.

[the Hep C elimination programme is] being done by the wrong people in that we haven’t. It’s being led by ODN, Operational Delivery Networks across the country and led by clinicians who haven't had any training in public health or screening or surveillance. (Participant 12)

Other interviewees felt it fitted within their remit to find new people to test and treat, especially as HCV is a curable disease, and that receiving the surveillance data accorded them a duty to contact those people, which motivated them to persist when faced with challenges.

#### Appropriateness of individual roles for the tasks (mixed)

Even if interviewees believed the exercise was rightly designated to their ODN, barriers could be faced at the individual role level because some tasks did not easily fit existing roles—particularly if clinical staff had to complete administration or data validation tasks. However, other interviewees saw the exercise as reflecting their role and thus they were able to prioritise it.

So we were very clear of who’s clinical and who’s nonclinical. So by us owning this, we knew what we had to do, where the nurses could get on and do whatever else they wanted. (Participant 13)

For these interviewees, the exercise coincided with other changes, resources or skills available to their ODN (see also environmental context and resources and skills domains).

### Skills

#### IT/data skills/screening training were needed (barrier)

Relating to the barriers reported in social/professional role and identity, some interviewees said that training in spreadsheets would have helped with the early data validation stage—both in terms of basic skills for spreadsheets/databases and in terms of the content of the surveillance data.

if you’ve got an IT geeky person that and I'm not, that knows how to do cross referencing of databases they could probably do things in a push of a button, as we're all just laboriously going through individual names case by case. (Participant 16)

Another participant said training in setting up public health screening programmes was needed to confirm the historical test results. Training may have helped reduce the barriers concerning the appropriateness of ODN and individual roles in the exercise. Unexpectedly, many interviewees did not feel that any general IT training would have been beneficial due to having ODN-specific systems and processes.

### Knowledge

#### There was a lack of up-to-date data about patients (barrier)

Another barrier related to data validation against laboratory records was a lack of up-to-date patient HCV status, given that the ODNs were using a static list from UKHSA. For example, a patient may have received treatment elsewhere since the re-engagement exercise started, but the ODN would not have received this update. An additional challenge that came to the fore later in the exercise, when contacting patients, was keeping up-to-date contact information.

With a lot of people, we could no longer find their… checking the NHS Spine [the personal demographics system]…can’t remember if it gave us addresses on the spreadsheet, but anyway we updated via the Spine to get more contemporaneous contact details with varying success (Participant 25)

The impacts of this knowledge gap are explored more in the Social Influences themes.

### Emotion

#### Stress and other negative emotions (barrier)

Due to the aforementioned barriers, many interviewees reported feeling stressed, overwhelmed and/or frustrated at times.

unfortunately when you get a spreadsheet with that amount of data on it is. It’s just overwhelming, really, to be honest with you. (Participant 16)

### Memory, attention and decision-making processes

#### The exercise required a lot of time and attention (barrier)

Another impact on individuals was that extensive effort and attention to detail was required.

I know I used to set myself a time. An hour every day to do the data scrutiny for weeks and weeks and weeks before we were ready to do the letters (Participant 11)

### Social influences: NHSE, UKHSA, ODNs, GPs

Throughout the exercise, interviewees were in contact with NHSE, UKHSA and other colleagues in their ODN.

#### Perceived pressure from UKHSA/NHSE to conduct the exercise (mixed)

Some interviewees perceived pressure, but also motivation, to complete the exercise because it was mandated by NHS England and UKHSA.

I think all ODNs had pressure to do the exercise because it was mandated by NHS England. So yeah, there was pressure to do it and we obviously wanted to do it in the best way that we could as well. (Participant 16)

It also caused some negative perceptions of pressure at times (see Emotion—Stress and other negative emotions). Other participants perceived no deadlines and no fixed protocol and were able to fit the project in among other priorities. However, they acknowledged that a lack of pressure may have reduced their focus and progress.

#### Interactions with UKHSA/NHSE during the exercise (mixed)

ODNs were told they could contact NHSE and UKHSA for support, from central and/or local field offices. Some meetings were held between ODN clinical leads and UKHSA during the re-engagement exercise. The interviewees who regarded these interactions with UKHSA and/or NHSE positively remarked on individual points of contact, such as local field services, and meetings that facilitated the exercise. Some interviewees said they were not satisfied with the support available from NHSE or UKHSA, stating that there was a lack of oversight of the project over time (especially during/after COVID-19) and that there was no feedback mechanism for errors in the surveillance data.

When NHS England put out a project they don’t never have a project plan, you know and you know they try their hardest to do what they need to do in the time they need to do it. But it’s not always a very clear message (Participant 6)

#### ODN was collaborative (facilitator)

Most participants felt that their colleagues in their ODN worked well together and coordinated decision-making, which enabled them to conduct the exercise.

our ODN is collaborative and there are good communication channels. (Participant 11)

#### Cooperation/support, or lack thereof, from GPs (mixed)

After validating laboratory data, when it came to sending letters to GPs, interviewees commonly observed a lack of response. Some interviewees viewed this as a hindrance to the exercise, whereas others described it as implicit consent to continue. A lack of response from GPs did lead to a standstill if the ODN could not make contact with patients directly. Some interviewees were reluctant to proceed without approval from the GP, indicating either lack of confidence in, or awareness of, the recommended procedure to continue without GP responses.

that’s really where it fell down because the GP practises didn’t have the time to sift through the amounts of stuff that we were sending them. So we were really at a bit of an impasse as to know whether it was appropriate to contact that person. So we left it and then we did the whole exercise again and we did get a few back. Few GPs did respond. (Participant 6)

Another issue resulting in an impasse was when GPs de-registered their patients, even though the patient had not moved. It was acknowledged that GPs and other practice staff were not all aware of the exercise and were already overburdened.

However, some interviewees reported that a small number of GPs, whom they had pre-existing working relationships with, helped support the exercise. Support included being the first point of contact for patients, writing referrals to the ODNs, offering testing in their practice, and informing patients of results. The interviewees from ODNs who did not return data to UKHSA more frequently reported a lack of response from GPs.

### Beliefs about consequences

#### Worried about people’s responses to the exercise (mixed)

Before starting to contact patients, interviewees anticipated potentially negative consequences. Concerns included the fear that they might accidentally disclose HCV status to unintended person/s, that patients would ask why they had not been notified earlier, or that patients would be unable to attend appointments (eg, because of transport costs).

The only thing that sort of we were a bit worried about in the beginning was the potential implications of someone kicking off getting a letter that they perhaps was incorrect. So that was a bit concerning for us (Participant 23)

The effects that these concerns had on the conduct of the exercise were mixed: they reportedly resulted in caution and delays starting, but also the implementation of additional strategies to reduce risks, which may have improved the outcome of the exercise (see more in Social influences - adaptations to patient protocols).

### Social influences: patients

#### It was difficult to make contact with people/patients (barrier)

After GPs were notified, ODN staff reported contacting patients, typically sending letters using or adapting the templates provided in the guidance. A lack of response to letters and phone calls from people/patients was commonly cited. Interviewees said they were unsure if they had failed to contact those people/patients or if they were declining treatment.

I would say at least half the attempts to contact the people probably never got to the individual, which is a big factor in all of this (Participant 23)

#### Most people/patients were happy to be contacted, but a small number of difficult conversations were had (mixed)

From patients they did make contact with, most interviewees reported a small number of difficult or distressed responses. Although infrequent, it was seen as an important barrier (see Beliefs about consequences*—*Worried about people’s responses to the exercise). Reported incidents included sending letters to the wrong address, unintended recipients reading the letter and people upset that the ODN was not aware that they had already been treated. There were also people upset if they could not reach the ODN (eg, because the phoneline was not operating that day/night/weekend).

One [patient phoned] to say “I’ve been treated in a trial. Why don’t you know that you shouldn’t have contacted me.” And the second one to say “this was delivered to me on a Friday and I couldn’t get through on the weekend and then the week after the [clinical nurse specialist] mobile wasn’t answered and I had a week of extreme anxiety” (Participant 25)

While most interviewees felt a responsibility to continue the exercise regardless, others reconsidered whether to continue, with an interviewee reporting stopping the exercise after a patient complaint.

Some interviewees reported that despite their anticipated concerns, most of the people they contacted were pleased to be contacted. These were said to be people who had some awareness of their diagnosis and were glad to have been contacted again, even if they had already been treated. Other people were happy once they had an opportunity to speak to someone at the ODN.

#### Effective adaptations to patient protocols (facilitators)

Interviewees reported several adaptations they made to their GP and patient contact strategies, which they associated with greater success in contacting and supporting patients. We coded these as facilitators resulting from social influences.

The adaptations included spending time deliberating over the language used in the letters such as not referring to hepatitis C, timing the sending of letters to days when support (eg, a phoneline) was available, or offering walk-in clinics.

what we agreed was anyone who was distressed could come into a… walk into a [day] clinic and just be tested then and there. So, or they were offered some reassurance. So that’s how we did it. We pretty much managed it, you know, really well at that point. (Participant 14)

Another interviewee said they included a blood booking form in the letter, enabling people to book at their local service. Additionally, some ODNs changed how/where they offered testing, so that people did not have to travel. This involved the use of mobile testing vans, which were partly brought about due to COVID-19 restrictions.

They [patients] love the fact that we go to them. And. They pick where we meet them. It amazed me at first. How many people let you park outside their house (Participant 22)

Other interviewees’ concerns that the methods of contact suggested in the exercise guidance would not be effective led them to adopt a more lengthy, multipronged contact protocol involving sending multiple letters and/or phone calls spaced out over weeks.

We decided to take a slightly different approach to it because we didn't think we were really gonna get the desired effect. I suppose by just sending a letter to both GPs and or patients, so we thought it would be better to kind of do a more hands on approach I think, as well as sending the letters, of course, but we want it to be a bit more. You know, I suppose going a bit deeper in terms of the communication we were gonna try. (Participant 1)

Another adaptation to protocols was to recontact the GP if they had not been able to contact a patient.

[after trying to contact patients] we’ll write to the GP and make them aware that we’ve tried everything we can. They’re not engaged. But if they want to be re-referred to the service we will happily offer them the next available appointment. I have had cases where once that letter was sent [to the GP], the patient received a copy of that letter and it actually spurred them to engage. (Participant 24)

### Beliefs about consequences

#### Treatment yield and other results from the exercise (mixed)

Towards the end of conducting the exercise, most interviewees acknowledged positive outcomes including treating patients who may not have received treatment at all or as early on.

we have actually had quite a good response in terms of the number of people we’ve been able to start on treatment (Participant 1)

Some interviewees also identified confirmation of negative PCR RNA test results as a beneficial outcome. However, some interviewees felt that the actual treatment yield was minimal, due to already knowing the patients, or because they were unable to contact them, which caused some to stop or deprioritise work on the exercise.

### Interviewee recommendations and lessons learnt for future re-engagement work

Interviewees were asked to reflect on their recommendations for further re-engagement work. These are summarised and mapped to intervention types^22^ in table 4. Suggestions included national campaigns informing the public, and/or engaging more with primary care practices, laboratories and other services to increase their awareness. In addition, national ODN meetings, more stakeholder consultation and specific objectives in a project standard operating procedure were seen as ways of improving the clarity and coordination of the project. If receiving further surveillance data in the future, interviewees requested the lists be pared down, be shared in smaller up-to-date batches, and be sent directly to individual hospital trusts or smaller regions/cities rather than the ODN hub. They also said that new national testing and treatment data and patient demographic data is available now that could be shared in more frequent, recent batches. They queried why there is no national live registry across all services for viral hepatitis, including drug and alcohol services that are operated by local councils, so that they can follow up people in their region. Interviewees also said they need ongoing appropriately resourced staff, including people with data analyst skills and peers with lived experience, the latter being vital for outreach purposes. Overall, there was a feeling that better tools are still needed for reaching people, and that more patient-centred approaches are needed to find and treat the people who have not been offered appropriate support into care.

**Table 4:** Themes of interviewee recommendations for future re-engagement work, mapped to intervention functions from the Behaviour change wheel^22^

Recommendation theme	Number of participants	Example quote	Intervention function for ODNs^22^
Surveillance data improvements/ODN data needs	7	“So, I suppose with the RNA results and I know UKHSA is in a much better position now in terms of understanding and being linked in with more labs and understanding kind of what the current or latest RNA results are for people. I think to be honest, if there had been an initial, that initial data cleanse, you know for treatment [of] patients, fine, I think that’s perfect for us to do that because that’s our sort of what we do.” (Participant 1)	Environmental restructuring
Enhance collaborations/partnerships with other sectors and services	9	“What would probably have been helpful would have been maybe to get into it with the GPs to say that this re-engagement exercise was happening. But I also know how difficult communication is with GPs at the moment. So yeah, I think if people have been more aware of what was happening, they may have—and why it was happening then we may have got more responses.” (Participant 9)	Education, Persuasion
Create shared programme for HCV elimination	3	“you’ve then got all these Hep C elimination initiatives that started in January 2020. It perhaps in hindsight would have been good to join it all up again. I mean I do think we're at the point now where if we’re strategic about it, you’ve got the mechanisms, you’ve got the GP champions. Do you know there would be a really good way to pull it all together now and see what’s left to be done.” (Participant 3)	Environmental restructuring
More staff/funding for further re-engagement work	9	“Potentially there could have been people that we could have reached if we had somebody on it full time and were kind of got on it a little bit faster that we might have been able to do it.” (Participant 1)	Incentivisation
Project management and national communication strategies	11	“But I do think a national meeting where everyone sat around and under… you know, even verbally was informed of what was happening, why it was happening, what to do with it… It was very much of here’s your spreadsheets away you go. Here’s some guidance notes, but I do think a more collaborative approach would have gone really well, but at the time I think we were all just very happy to receive that list” (Participant 14)	Education, Persuasion, Training
Use more patient-centred care approaches and communication methods	10	“Our whole kind of focus, our whole kind of what’s been sort of deemed acceptable practice has changed an awful lot over this time. So you know, it was a very paternalistic model of care. Probably when we started the programming that patients you know had to come and see us and now for some patients, we’re actually going to their doors and providing treatment on site. So you know it’s a learning curve in that regard” (Participant 12)	Training

GPs, general practitioners; HCV, hepatitis C virus; ODNs, Operational Delivery Networks; UKHSA, UK Health Security Agency.

Finally, table 5 summarises the other re-engagement approaches ODNs have used since the re-engagement exercise began and the advantages of these, which could be built on.

**Table 5:** Themes of other re-engagement approaches being used by ODNs

Other re-engagement approach themes	Number of participants	Example quote
Working with drug and alcohol or prison services	2	“we’ve done a lot of work with drug and alcohol services to screen in those services and re-engage and people who are known to be positive with hepatitis C, both you, you know many ways but not least by sort of going out to the drug and alcohol services and running clinics on site and so on” (Participant 12)
Within ODN outreach methods: employing find and treat/patient coordinator roles and running testing events with incentives	4	“Our re-engagement comes from our testing projects because we test prolifically across the [region] and that’s where we pick up a lot of our re-engagement stuff. You know, so there’s lots of people that get tested. We incentivise really well. So people come along and because of the incentives they get tested and then we actually find out that you know they've been on our books for some time and we reengage with them” (Participant 6)
Working with primary care	6	“we’ve got some work going on with GP practices at the moment around the region to try and more proactively work so with them such that they they’re then given. Their patient list to proactively go and try and get them in, and they're incentivised for that.” (Participant 23)
Re-engaging patients when picked up in the emergency department	1	“all of a sudden they popped up again in ED [Emergency Department] and it potentially gives us an opportunity and in to give them a ring or if we get it quick enough, we can potentially see them on the ward if they're an inpatient. So that’s been quite good from a re-engagement point of view.” (Participant 1)
Employing lived experience peers (from the Hepatitis C Trust)	7	“If the [patient is] not engaged and I will obviously reach out [to] the Hep C trust to see if they can try and track them down. We've got a great bunch of peers who are really. They’re great with the patients, they build up rapport with many of our regulars.” (Participant 24)

GP, general practitioner; ODNs, Operational Delivery Networks.

Most of these approaches involve partnering with other services and charities, as well as localised community testing delivery. Some also commented that the people who still have not re-engaged with care are likely to have more complex needs and have faced stigma or discrimination; the Hepatitis C Trust peer programme (entailing personalised approaches to engaging individuals by building a relationship and supporting them to attend testing or receive tests at home) was cited as vital to working with this population. One interviewee cited the Emergency Department Opt-Out testing programme (piloted at sites in England with very high HIV prevalence^23^), where people testing positive are quickly linked (back) to the ODN. Finally, furthering partnerships with GPs was also progressing for some ODNs, with incentivisation, addressing logistical barriers in GP practices, and fostering stronger relationships between ODNs and GPs through the recent NHSE GP Champions initiative, who are employed to conduct HCV patient case-finding in practices around England.

## Discussion

Key barriers occurred in environmental context and resources (eg, staffing, interruptions due to COVID-19 and mixed laboratory access), and social influences—particularly uncertainty about how to respond to limited responses from GPs and difficulty contacting patients, with a small number of patients unhappy to have been contacted. Interviewees also reported difficulty with the stress and level of attention/detail required (emotion, memory, attention and decision-making domains) due to the volume of data they received. Beliefs were mixed about the yield from the exercise and whether it was appropriate for ODNs and individual staff to be assigned the work, although they commonly reported some positive outcomes and lessons learnt. On the positive side, facilitators included ODN goals and NHS targets aligning with the exercise, an overall positive response from patients, and ODN specific (regional) resources, systems and established relationships with laboratories and GPs. Interviewees also reported adaptations to the exercise that enhanced patient support and that they associated with greater success in making contact. Interviewees’ recommendations for future re-engagement work emphasised enhanced processes, resources and infrastructure for partnering with other services and outreach partners to find and re-engage people into care.

WHO Europe published the NHSE-UKHSA re-engagement exercise as an example of best practice^5^ and this qualitative evaluation contributes insights into building on the re-engagement exercise successes should it be considered again, or attempted in another setting or country. Interestingly, the delivery of this exercise was structured according to the NHS health services in England and yet achieved a similar yield to the nationally rolled out retrieval exercise in the Netherlands,^11^ and a similar exercise by NHS Wales.^15^ In the Netherlands, they reported greater success in their smaller regional trial,^12^ and in this exercise, ODNs also reported regional and environmental influences (eg, access to laboratories, staff availability, the varied impact of COVID-19 and local adaptations to patient contact and support). Regional resources and processes may explain the variation in return of data to UKHSA and the exercise outcomes.^6^ Key lessons to be learnt, if applying this approach to other settings or infectious diseases, would be the need for regionally commissioned and tailored approaches with sufficient resource and training to support data validation, and the use of outreach or patient-centred care methods to supplement any patient letter mailing approaches.

This qualitative evaluation also contributes to other theory-based qualitative studies on hepatitis C, using the TDF and a similar framework, the COM-B model^22^ of behaviour change (Capability, Opportunity, Motivation: Behaviour; similar but less specific than the TDF). For example, Ruiz *et al*^17^ used the TDF to analyse barriers and facilitators to an opt-out HCV testing programme in prisons and found the main barriers in environmental context and resources (eg, organisational setting restrictions and human resources), and social influence domains (getting other parties’ cooperation). Whiteley *et al*^24^ investigated provider-related barriers and enablers to a new HCV treatment pathway in primary care, using the COM-B model. They found barriers, for example, in the domains: motivation (attention/decision-making in TDF) and opportunity (environmental resources/social influences in TDF). Fontaine *et al*^19^ explored barriers and facilitators in HCV testing among people who inject drugs and service providers, and the main themes found with service providers were on knowledge, skills and confidence, professional roles, resources and integration of services, social and emotional factors and behavioural regulation and incentives for HCV testing. This re-engagement exercise qualitative evaluation adds to this body of work demonstrating that a theory-based approach using the TDF can provide evidence for the effectiveness of programme implementations and which domains need to be addressed in interventions.

### Strengths and limitations

Strengths of this study include the use of a theory-driven framework for comprehensive analysis of barriers/facilitators. The sample was well distributed across our sampling frame, but this evaluation does not represent all ODNs and individual staff views. There was no cut-off date for the exercise and there was variation in how ODNs approached the task. This results in some ambiguity in assessing the barriers and facilitators at a consistent time point. We also acknowledge recall limitations given the passage of time since the exercise began, which was particularly an issue for interviewees’ recall of the original exercise guidance. For the same reason, we did not include patient perspectives in this study, as the amount of time passed would limit our ability to adequately recruit patients who were contacted during the re-engagement exercise. Because it is important to understand patient perspectives, we are also conducting a stand-alone patient interview study about factors affecting their re-engagement in care after a period of disengagement.

### Implications

Qualitative evaluations provide insight into how to improve on the successes and overcome some of the barriers from the re-engagement exercise. Drawn from the analysis, good practice can be enhanced through (1) improvements to surveillance data quality and linkage, including centralised systems that enable sharing more up-to-date (non-static) patient information and guidelines on how to feed back about data. In addition, consultations with care providers on their data and resource needs should be carried out. (2) Provide resources to care providers to complete pieces of re-engagement work within time-funded posts and provide training and support as needed (eg, with data sharing agreements, data analysis/spreadsheets, population screening). ODNs have developed successful regional approaches to re-engagement; therefore, it is important to further support and fund regionally tailored re-engagement work. (3) Include patient perspectives and evidence-based approaches to communication and service delivery (eg, letters may be more effective than phone calls,^25^ letters including a blood test booking form are more effective^26^). In this evaluation, interviewees reported successful attempts to reduce patient distress by providing more in-person and telephone support, as well as outreach services. Fears of patient complaints have been found in other trials,^27^ but the exercise was more acceptable to patients than some anticipated. Focused communication strategies^28^ may also help people who have declined treatment due to facing stigma and discrimination, those who are not aware of the newer, more effective cures, and those who need additional health services before treatment.

To enable these improvements to take place, funding and dedicated resources are vital to ensuring the sustainability of re-engagement initiatives, including joint efforts with other disease elimination strategies.^29^

### Conclusions

This qualitative evaluation of the re-engagement exercise has identified important contributions to evidence and public health practice on the re-engagement of historically diagnosed patients who have been lost to follow-up. Very few such initiatives have been attempted globally, and qualitative evaluation is vital to investigating the barriers, facilitators and lessons that can be learnt. ODN interviewees expressed strong commitment to the aims of the exercise. Patients have been treated who may not have been at all, or as early in the progression of their disease. These are important accomplishments and good examples of how sharing public health surveillance data can work with health services to deliver on global health strategies. We have drawn out the key lessons to be learnt and shared from this evaluation so that global efforts can continue to build on the progress towards re-engaging patients and eliminating viral hepatitis by 2030.

## Supplementary material

10.1136/bmjopen-2025-104546 online supplemental file 1

10.1136/bmjopen-2025-104546 online supplemental file 2

10.1136/bmjopen-2025-104546 online supplemental file 3

## Data Availability

Data are available on reasonable request.
