# Supplementary material for: Qualitative evaluation of the barriers and facilitators to a retrospective hepatitis C virus patient re-engagement exercise in England
Source: BMJ Open. 2025 Nov 13;15(11):e104546. doi: 10.1136/bmjopen-2025-104546 (PMC12625882; doi:10.1136/bmjopen-2025-104546)
Supplement: online supplemental file 1 [file bmjopen-15-11-s001.docx]

### **Topic Guide for Re-engagement Exercise Evaluation**

Thank you for taking the time to speak to me today. I am [name, job title and organisation].

This will take about 45 mins to an hour. There are no right or wrong answers. Any questions before we proceed?

**1) About them.**

1. Can you tell me your understanding of the aim of the re-engagement exercise? [knowledge]
2. How was it conducted in your ODN?

- Prompt: elaborate on data validation, contacting GPs, contacting patients
- Prompt: How did your ODN decide which patients to contact?

1. What role did you play in the re-engagement exercise? [Social/ Professional Role and Identity]

Prompt for details: when they were involved, what they did, etc

1. Who was responsible for conducting which aspects of the exercise? [Social/ Professional Role and Identity]

**2) Barriers and facilitators.**

I would now like to ask some questions to learn more about potential factors that may have made it easier or harder to do the re-engagement exercise.

**2a) Training, responsibility and resources.**

First, I would like to hear about your views of the training, responsibilities and resources of your ODN during the exercise.

1. Did your ODN receive any guidance for conducting the re-engagement exercise? [Knowledge]
   - Prompt: Who from (e.g. from PHE/UKHSA), was it sufficient?
2. In your opinion, did your ODN have sufficient training to carry out the exercise? [Skills]
3. Would it have been helpful to have received some training or guidance? [Skills]
   - Prompt for details of what type of training/guidance
4. What strategies or ways of working within your ODN do you have in place that helped you before you started the exercise? [Behavioural Regulation]
5. Did your ODN have any action plans for how to approach the exercise? [Behavioural regulation]
6. To what extent did you feel it was part of your ODNs responsibility to conduct the re-engagement exercise? Who else was/should have been responsible? [Social/ Professional Role and Identity]
7. Did the exercise require changes to the roles and responsibilities of any team members, including yourself? [Social/ Professional Role and Identity]
8. To what extent did you feel that your ODN had sufficient resources? [Environmental Context and Resources]

- Prompt: Time, Staff, information, other resources

1. Are there any additional resources you needed to conduct the re-engagement exercise? Were these available? Who was responsible for making them available? [Environmental Context and Resources]
2. To what extent did COVID19 impact on the exercise? [Environmental Context and Resources]
   - Prompts: In what ways (e.g., were staff redeployed)? When (if at all) have you been able to resume as before, or is it different still? Did anything good come out of it?
3. What strategies did your ODN use to overcome any challenges? [Behavioural Regulation]

**2b) The impact of the exercise.**

I would now like to know more about your impressions of the impact of the exercise.

1. Were there any incentives in place for your ODN to conduct the re-engagement exercise? [Reinforcement]
2. Were there any benefits of conducting the exercise? What about any downsides? [Reinforcement or Beliefs about Consequences]
3. To what extent do you feel it was necessary to conduct the re-engagement exercise? [Intention]
4. Had the exercise not been conducted, what do you think the consequences would be? [Beliefs about consequences]
5. In your opinion, how much of a priority was the re-engagement exercise in your ODN? [Goals]
6. Did your ODN have any targets related to the re-engagement exercise? [Goals]
   - Prompt: what were they? Who were they set by? How realistic were they?

**2c) Inter-organizational influences on the exercise.**

The next questions are about your views on interactions with external partners and other influences on the exercise.

1. Did your ODN interact with local laboratories? What influence did these interactions have on conducting the exercise? [Social influences]
2. To what extent were GPs involved in the exercise? [Social influences]
   - Prompt: what did they do? Were some GPs more involved than others? What impact did their involvement have?
3. How did patients react to the re-engagement exercise? [Social influences]
   - Prompt: how did they react to contact attempts? What method of contact was more successful? Was the method appropriate? How did they feel about coming for testing? And treatments?
   - Prompt: What did you do if patients were not engaging?
4. How were colleagues/staff in your ODN working together during the exercise? What influence did this have on the exercise? [Social influences]
5. Did your ODN interact with PHE/UKHSA during the exercise? What influence did these interactions have on conducting the exercise? [Social influences]
   - Did you want more interaction? e.g. what about UKHSA local field services? Why/why not?
   - Did you give them feedback on the exercise?
6. To what extent did you find it easy or difficult to return a patient outcomes list to UKHSA? [Behavioural regulation] (if they are unsure what you mean, describe that there was a template spreadsheet)

**2d) Personal factors and emotions.**

I would like to learn more about your personal experiences with the exercise.

1. How motivated were you personally to complete the exercise? Did that change over the course of the exercise? [Intention]
2. To what extent did you feel pressured to complete it? [Emotion]
3. How much of your attention did the re-engagement exercise require? [Memory, Attention and Decision-Making Processes]
4. How confident did you feel in your ability to conduct the re-engagement exercise? [Beliefs about Capabilities]
5. Were you optimistic or pessimistic that conducting the re-engagement exercise would help re-engage patients in care? Why? [Optimism]
6. Did you have any worries or concerns about the re-engagement exercise? [Emotion]
7. Was there anything you found personally rewarding about conducting the exercise? [Reinforcement or Beliefs about Consequences]

**3) Other approaches to reengaging patients into care.**

1. Other than the PHE/UKHSA re-engagement exercise, has your ODN undertaken any other re-engagement activities - in the past and/or currently? If so, how did those activities differ from the PHE/UKHSA exercise?

Prompt: what was done, who was involved, what resources or support, how effective was it in comparison? Did you face any different challenges/barriers?

**4) Final comments**

1. Anything else you would like to add or reflect on?
2. If we wanted to do the re-engagement again, what changes in your ODN would be needed? [behavioural regulation]
3. (optional): ask if they would be happy to refer GPs and patients to us for further interviews.
