# Supplementary material for: Qualitative evaluation of the barriers and facilitators to a retrospective hepatitis C virus patient re-engagement exercise in England
Source: BMJ Open. 2025 Nov 13;15(11):e104546. doi: 10.1136/bmjopen-2025-104546 (PMC12625882; doi:10.1136/bmjopen-2025-104546)
Supplement: online supplemental file 2 [file bmjopen-15-11-s002.docx]

**General approach/decisions**

Basic coding labels:

- Create a code under the best fitting domain, in words that summarise what the participant says – in their words, not according to any interpretation at this stage.
- At the end of the code, label whether it appears to be a barrier (b) or facilitator (f) – if unsure you can leave this out for now.

Broad/narrow coding:

- If they mention several domains in one sentence, break the sentence down as much as possible.
- Data can overlap across domains but be careful that it doesn’t happen too often.

Temporal issues:

- For statements of what they’d need if they were to repeat the exercise in the future, keep it outside the TDF, instead code to Recommendations
- Some ODNs have continued doing re-engagement work using the original lists, or merged with their own lists, and encountered various barriers and facilitators. Even if these occurred after the original exercise, still code them as barriers and facilitators in the main analysis, as there wasn’t really a clear cut off date for the exercise and there was variation in how ODNs approached the task.
  - - If they are referring to a very different re-engagement activity (that did not include contacting patients from lists of pooled data), then do not include this in the TDF coding, unless they’ve mentioned that the additional reengagement exercise was in response to a barrier they encountered. Code these different re-engagement activities under the Recommendations code tree, to be later collated.

Recall issues and contradictory statements:

Due to the passage of time participants recall the way they carried out the re-engagement exercise, and the way the project was managed, differently from other participants.

- We do not need to address this in the coding phase.
- In the write up, acknowledge recall bias or perhaps outdated procedures, that different accounts exist.
  - wide variety between ODNs and even within ODNs
  - Possible recommendation down the line could be for the guidance to encourage consistency.
  - Whilst ODNs liked the freedom to do it their own way, it could be that some elements could be standardized whilst others remain more open.

**Distinguish between:**

1) If they provide a simple description of what they did, without reference to a facilitator or barrier, this does not need to be coded

E,g. when they ‘always sent letters no phoning’ – this wasn’t because they encountered a facilitator/barrier, but just because that was the guidance. And they confirmed that they followed the guidance procedure.

2) Separately code a separate adaption/change implemented by the ODN to address a perceived barrier, like ‘changed procedure to suit GPs’

**TDF domains, definitions, and examples**

| **Domain** | **Construct** | **Decision rule** | **Examples** |
| --- | --- | --- | --- |
| 1. **Knowledge**   *An awareness of the existence of something* | **Knowledge (including knowledge of condition/scientific rationale):** An awareness of the existence of something  **Procedural knowledge:** Knowing how to do something  **Knowledge of task environment:** Knowledge of the social and material context in which a task is undertaken. | Consider coding to this domain:  Awareness of the aims of the re-engagement exercise  Awareness/knowledge of issues associated with the surveillance data  Procedural knowledge: awareness of how to conduct the re-engagement exercise  Awareness of (progress towards) HCV elimination efforts  Inappropriate coding to this domain:  Reasons for following/not following procedures  Their level of ability to carry out the procedures – code to skills  Knowledge benefits from conducting the exercise - code to beliefs about consequences  Knowledge/awareness of patients needs – code to Social influences | Of course it changes because you know when we got that data, it was in 2017. So a lot of those, you know, it changes again, doesn't it? That data is it's out of date. Very quickly, but we managed to get it all done. (ppt 4, barrier- currency of data poses a challenge)  also what was erroneous with some of those is the samples were taken within the 1st 12 months, which is if you know and think about hepatitis C is too soon to know because it can just be as a product of still can be the mother's antibodies because it's so close to delivery and birth.  (ppt 11, barrier- knowledge gap error in data data) |
| 1. **Skills**   *An ability or proficiency acquired through practice* | **Skills:** An ability or proficiency acquired through training and/or practice  **Skills development:** The gradual acquisition or advancement through progressive stages of an ability or proficiency acquired through training and practice  **Competence:** One’s repertoire of skills, and ability especially as it is applied to a task or set of tasks  **Ability:** Competence or capacity to perform a physical or mental act. Ability may be either unlearned or acquired by education and practice  **Interpersonal skills:** An aptitude enabling a person to carry on effective relationships with others, such as an ability to cooperate, to assume appropriate social responsibilities or to exhibit adequate flexibility  **Practice:** Repetition of an act, behaviour, or series of activities, often to improve performance or acquire a skill  **Skills assessment:** A judgement of the quality, worth, importance. Level or value of an ability or proficiency acquired through training and practice | Consider coding to this domain:  Any courses or training they received or would like to have received.  Skills and experience that they had while conducting exercise.  Inappropriate coding to this domain:  Descriptions of how easy/difficult it is to do the exercise - Consider coding to Beliefs about Capabilities  Descriptions of how confident they are – Consider coding to Beliefs about Capabilities | I was already working in [department] for a long time I was a secretary there. So I knew the systems (ppt 4) |
| 1. **Social/professional role and identity**   *A coherent set of behaviours and displayed personal qualities of an individual in a social or work setting* | **Professional identity:** The characteristics by which an individual is recognised relating to, connected with or befitting a particular profession  **Professional role:** The behaviour considered appropriate for a particular kind of work or social position  **Social identity:** The set of behavioural or personal characteristics by which an individual is recognizable [and portrays] as a member of a social group  **Identity:** An individual’s sense of self defined by a) a set of physical and psychological characteristics that is not wholly shared with any other person and b) a range of social and interpersonal affiliations (e.g., ethnicity) and social roles.  **Professional boundaries:** The bounds or limits relating to, or connected with a particular profession or calling  **Professional confidence:** an individual’s belief in his or her repertoire of skills and ability especially as it is applied to a task or set of tasks.  Group identity: the set of behavioural or personal characteristics by which an individual is recognizable [and portrays] as a member of a group  **Leadership:** The processes involved in leading others, including organising, directing, coordinating and motivating their efforts toward achievement of certain group or organization goals  **Organizational commitment:** An employee’s dedication to an organisation and wish to remain part of it. Organisational commitment is often described as having both an emotional or moral element and a more prudent element | Consider coding to this domain:  What role they brought to the exercise  Discussion about how the reengagement exercise fits with their normal role  The extent to which it was a change in their role, or the role of colleagues/ODN  Whether they felt it was their responsibility to carry out the exercise (or who else should have been responsible)  Discussion of how they define themselves or any characteristics or roles they have which are relevant to their behaviour at work/the re-engagement exercise  Their moral stance towards patient care  Organisational commitment  Inappropriate coding to this domain:  Discussion of support from their profession or colleagues – Consider coding to Social Influences  Self-confidence in their ability to conduct the exercise – consider beliefs about capabilities | I was the middle man that you know for the whole thing so because I wore all those different hats anyway, so you know it was quite smooth running (ppt 4)  on occasion I had to answer the helpline. I know that wasn't the plan...It should have been a nurse if it just so happened. Sometimes I would have to answer the helpline and I would take information, but then the nurse. Would call back.  ...it wasn't it was those supposed to speak to somebody clinical, not to an admin person?  Interviewer  And that in a way that was a change in your role then, right? Because that was something that you actually would never be supposed to do.  Participant 11  I do speak to patients, but these, of course, weren't patients. These were people who were getting a letter...  So it was, yeah, slightly less comfortable (ppt 11)  But we felt we had to do everything that we could... morally to try and engage that patient and then to make sure that we're left kind of like a legacy of, you know, legacy may be the wrong word to suppose that well maybe like a trail... We’d left some… an imprint on their record so that if a clinician came into contact with them in the future, they would be able to offer them, that patient a test or you know… did that make sense? (ppt 14) |
| 1. **Beliefs about capabilities**   *Acceptance of the truth, reality, or validity about an ability, talent or facility that a person can put to constructive use* | **Self-confidence:** Self-assurance or trust in one’s own abilities, capabilities and judgement  **Perceived competence:** An individual’s belief in her or her ability to learn and execute skills  **Self-efficacy:** An individual’s capacity to act effectively to bring about desired results, as perceived by the individual  **Perceived behavioural control:** an individual’s perception of the ease or difficulty of performing the behaviour of interest  **Beliefs:** The thing believed; the proposition or set of propositions held true  **Self-esteem:** The degree to which the qualities and characteristics contained in one’s self concept are perceived to be positive  **Empowerment:** The promotion of the skills, knowledge and confidence necessary to take great control of one’s life as in certain educational or social schemes; the delegation of increase decision-making powers to individuals or groups in a society or organization  **Professional confidence:** An individual’s beliefs in his or her repertoire of skills, and ability, especially as it is applied to a task or set of tasks | Consider coding to this domain:  Descriptions of how easy or difficult it was for them to carry out the exercise   - The data validation - GP contact - Patient contact   Descriptions of how confident a participant felt in carrying out the exercise and why  Traits they have that enable/prevent them from doing the exercise  Other people’s confidence in them  Inappropriate coding to this domain: | I mean, I've been doing this job since now. So yeah, actually even more confident now.  It was all quite new to me at the time. (ppt 4)  once I started I had very little overview from anyone else that I was doing it correct. You know, it was just this was the process and.  ...They [ODN leads] seem to trust how I work with data, so I've got a track record of how I work with data and what I do so I can only assume that they trust and feel confident that I was going to do it (ppt 11) |
| 1. **Optimism**   *The confidence that things will happen for the best or that desired goals will be attained* | **Optimism:** The attitude that outcomes will be positive and that people’s wishes or aims will be ultimately fulfilled  **Pessimism:** The attitude that things will go wrong and that people’s wishes or aims are unlikely to be fulfilled  **Unrealistic optimism:** the inert tendency for humans to over-rate their own abilities and chances of positive outcomes compared to those of other people | Consider coding to this domain:  Think of this domain as ‘bigger picture’ optimism  Statements of their levels of optimism that they will re-engage patients via this exercise  -Such as believing that the exercise would help them reach elimination.  Pessimism – where they perceived that the exercise would not help them re-engage people at all  Changes in optimism/pessimism during the exercise  ***Code both positive and negative answers***  Inappropriate coding to this domain:  Beliefs about the outcomes/consequences of the exercise that they achieved.  Mentions of punishments/incentives/rewards received/hoped to receive– code to reinforcement | I would say I would err towards pessimistic because of just think we do...We’ve done similar things to patients, so we write to patients who have been referred to us who we know have got hepatitis C who are RNA positive, who don’t engage with us ....So I knew that that we probably weren’t going to get much from this. (ppt 11, barrier – pessimistic from the onset)  Yeah, I mean, but these are a hard to re-engage groups, you know, by definition. But very optimistic that there was now a process in place to at least try (participant 0) |
| 1. **Beliefs about consequences**   *Acceptance of the truth, reality or validity about outcomes of a behaviour in a given situation* | **Beliefs:** The thing believed; the proposition or set of propositions held true  **Outcome expectancies:** Cognitive, emotional, behavioural, and affective outcomes that are assumed to be associated with future or intended behaviour. These assumed outcomes can either promote or inhibit future behaviours.  **Characteristics of outcome expectancies:** Characteristics of the cognitive, emotional and behavioural outcomes that individuals believe are associated with future or intended behaviours and that are believed to either promote or inhibit these behaviours. These include whether they are sanctions/rewards, proximal/distal, valued/not valued, probable/improbable. Salient/not salient, perceived risks or threats.  **Anticipated regret:** A sense of the potential negative consequences of a decision that influences the choice made: for example an individual may decide not to make an investment because of the feelings associated with an imagined loss    **Consequents:** An outcome behaviour in a given situation | Consider coding to this domain:  **Prior expectations** about stakeholder reactions when starting the exercise and how these expectations influence ongoing conduct of the exercise  Anticipated regrets/  Beliefs about the consequences of (not) conducting the exercise    Beliefs about why they had the results/outcomes that they did (e.g. achievements/non achievements)  Beliefs about why the outcomes have been beneficial to the ODN    Change of knowledge/perceptions during exercise that relate to their outcomes.  ***Code both positive and negative answers***  Inappropriate coding to this domain:  During exercise: How stakeholder interactions have influenced conducting of the exercise – consider Social influences    Concrete rewards/incentives/punishments that encourage them to continue/do exercise again– code to reinforcement | Interviewer  if it had not been conducted, what do you think the consequences would have been?  Participant 11  Handful of people wouldn’t have been treated. I think it’s literally a handful. (ppt 11)  Yeah. I did [feel beneficial to do the exercise] and although I can’t find anything that actually says the numbers, I’ve got a feeling around about 10%, we had about 10% uptake, which is pretty good and that that was [my hospital]. So that was quite yeah, you know that was really good. I mean, I know [ODN lead] was pleased with it.  (ppt4) |
| 1. **Reinforcement**   *Increasing the probability of a response by arranging a dependent relationship, or contingency, between the response and a given stimulus* | **Rewards (proximal/distal, valued/ not valued, probable/improbable):** Return or recompense made to, or received by a person contingent on some performance  **Incentives:** An external stimulus, such as condition or object, that enhances or serves as a motive for behaviour  **Punishment**: The process in which the relationship between as response and some stimulus or circumstance results in the response becoming less probable; a painful, unwanted or undesired event or circumstance imposed as a penalty on a wrongdoer  **Contingencies:** A conditional probabilistic relation between two events. Contingencies may be arranged via dependencies or they may emerge by accident  **Sanctions:** A punishment or other coercive measure, usually administered by a recognized authority that is used to penalise and deter inappropriate or unauthorized actions. | Consider coding to this domain:  Incentives and benefits resulting from the exercise, or lack of  Any punishment/sanction resulting from completing/not completing the exercise  Consider:   - Financial incentives - Praise - Past experiences - Intrinsic (explicitly stated) rewards - Punishment   Inappropriate coding to this domain:  Discussion of social reinforcement   - Consider coding to Social Influences   Beliefs about consequences/outcomes even if these are said to reinforce future behaviour – Code to beliefs about consequences    Generally feeling pleased/unhappy – consider Emotion  Believing they would/n’t be successful in this exercise due to past experiences – code to Optimism/Pessimism | And obviously we apologised about [sending a letter to a child] and I think [clinical lead] had to speak to the parents on that and I obviously got a wrap on the knuckles because I should have checked, but that is one of the things that if we’re gonna do it again, it would be a good thing to highlight to people...(ppt 4, punishment)  Yeah, I did [find it personally rewarding]. Because you get people treated, people that didn’t know they had the virus… We’re working on stuff now and it’s great when you actually get somebody through MDT to treatment with something that could potentially kill them. You know. So, gosh. Yeah, it’s very rewarding doing it on my part. (ppt4, Intrinsic reward) |
| 1. **Intention**   *A conscious decision to perform a behaviour or a resolve to act in a certain way* | **Stability of intentions:** ability of one’s resolve to remain in spite of disturbing influences  **Stages of Change model:** A model that proposes that behaviour change is accomplished through five specific stages  **Transtheoretical model and stages of change:** a five-stage theory to explain changes in people’s health behaviour. It suggests that change takes time, that different interventions are effective at different stages, and that there are multiple outcomes occurring across the stages | Consider coding to this domain:  Descriptions of their mental resolve to conduct the exercise  Descriptions of what might make others more or less resolved to conduct the re-engagement exercise  Whether intention changed over time  Whether they would want to do the exercise again in the future  Inappropriate coding to this domain:  Perceived necessity of the exercise in order to achieve outcomes (‘Beliefs About Consequences’)  How they prioritised it or wanted it to end (‘Goals’).  Be careful not to code the reasons for the intention (focus on statements that directly reflect their intention)  References to their job/role containing a duty to carry out their job in a certain way (‘Social-Professional Role and Identity’) | So, kind of like… kind of just giving above and beyond what we thought was needed. (ppt 14)  No, we were committed. It was committed to that letter and doing it this way. Yeah. (ppt11) |
| 1. **Goals**   *Mental representations of outcomes or end states that an individual wants to achieve* | **Goals (distal/proximal):** Desired state of affairs of a person or system, these may be closer (proximal) or further away (distal)  **Goal priority:** Order of importance or urgency of end state toward which one is striving  **Goal/target setting:** A process that establishes specific time-based behavioural targets that are measurable, achievable and realistic  **Goals (autonomous/controlled):** The end state toward which one is striving: the purpose of an activity or endeavour. It can be identified by observing that a person ceases or changes their behaviour upon attaining this state; proficiency in a task to be achieved within a set period of time.  **Implementation intention:** The plan that one creates in advance of when, where and how one will enact a behaviour | Consider coding to this domain:  The priority of the re-engagement exercise for the ODN/individual  Any targets they set themselves or how they tried to reach targets set by others  The high level process of creating their planned goals    A state they’ve achieved where they may have stopped the exercise or do not want to do it again because they achieved all they wanted to  Desire to reach HCV elimination goals (that relate to the re-engagement exercise)  Inappropriate coding to this domain:  Clear examples of action planning – Consider coding to Behavioural Regulation  Pressures from others – code to Social influences | we were given a timescale of so many months in which we had to process and use this data. That was a CQUIN target because the ODNS are all part of a CQUIN, which is an NHS care quality health innovation.  …and we get funding. So you have to you have to meet CQUIN targets and CQUIN metrics. (ppt 11)  we wanted to make sure that we were contacting people in the right way and making sure we can get them back in. So we did take a probably a slower approach maybe to some other areas (participant 1)  yeah, we kind of left it open-ended and we didn't. I didn't get the impression that there was, that the project, the exercise itself had a particular target. Maybe that would have help- I don't know. Yeah. But again, I guess that it would be very difficult for them to put a target without them providing any kind of assistance. (participant 18) |
| 1. **Memory, attention and decision processes**   *The ability to retain information, focus selectively on aspects of the environment and choose between two or more alternatives* | **Memory:** The ability to retain information or a representation of a past experience, based on the mental processes of learning or encoding retention across some interval of time, and retrieval or reactivation of the memory; specific information of a specific task  **Attention:** A state of awareness in which the senses are focussed selectively on aspects of the environment and the central nervous system is in a state of readiness to respond to stimuli  **Attention control:** The extent to which a person can concentrate on relevant cues and ignore all irrelevant cues in a given situation  **Decision making:** The cognitive process of choosing between two or more alternatives, ranging from the relatively clear-cut to the complex  **Cognitive overload/tiredness:** The situation in which the demands placed on a person by mental work are greater than a person’s mental abilities | Consider coding to this domain:  Discussion about how frequently participant did the exercise  Discussion about how often the participant is made aware of the exercise  Discussion about the process of weighing up the pros and cons of how they went about conducting the exercise  How they made decisions  The mental effort required and fatigue/tiredness of the exercise  Inappropriate coding to this domain:  General awareness of re-engagement exercise aims and requirements – Consider coding to Knowledge  What the pros and cons of the exercise were – consider coding to Reinforcement or Beliefs about consequences | It was full on. I know that it was and it took up a lot of my time**…**  Well, it did. I mean, yeah, including my other work, and it all had to be so…so it was fiddly. It wasn’t difficult, but it was just a bit fiddly because there was so much data, you know? (ppt 4)  I think as with any kind of project, I suppose the longer it goes on, more projects kind of pile on, so it's harder to sort of maintain entire focus on it. (participant 1) |
| 1. **Environmental context and resources**   *Any circumstance of a person’s situation or environment that discourages or encourages the development of skills and abilities, independence, social competence, and adaptive behaviour*  *(opportunity)* | **Environmental stressors:** External factors in the environment that cause stress  **Resources/material resources:** Commodities and human resources used in enacting a behaviour    **Salient events/critical incidents:** Occurrences that one judges to be distinctive, prominent or otherwise significant  **Person x environment interaction:** Interplay between the individual and their surroundings  **Barriers and facilitators:** In psychological contexts, barriers/facilitators are mental, emotional or behavioural limitations/strengths in individuals or groups | Consider coding to this domain:  Resources and access to them e.g. staff, time, data  External constraints on them/their ODN  Events such as COVID affecting the environment  ODN financial concerns  The individual being affected by their environment  Inappropriate coding to this domain:  Discussion around how much of a priority the exercise was during COVID – consider Goals  Attributions of social pressure/interactions- Consider coding to Social Influences | So I went to [city]. Did that. Did probably three quarters of their letters and COVID hit and that was it. And although I have access, I can sit here and have access to [hospital] and [hospital], I can’t actually type letters from here, so I actually had to be... (ppt4)  …was a delay in the [name] hospital in [city] … everything had to go through their governance. And it took ages and ages. It took months for anything to go through governance, and then they’d do something and then they would change a letter that then had to go through governance. So there’s often a delay … some hospitals have got barriers, which it ends up being quite detrimental to patients.(ppt 4)  we’ve got a our hospital system is excellent and once you’ve got the letter set up and we’ve got the configuration correct and we’ve got the right envelopes, it wasn’t a problem, (ppt 11) |
| 1. **Social influences**   *Those interpersonal processes that can cause individuals to change their thoughts, feelings, or behaviours* | **Social pressure:** the exertion of influence on a person or group by another person or group  **Social norms:** Socially determined consensual standards that indicate a) what behaviours are considered typical in a given context and b) what behaviours are considered proper in the context  **Group conformity:** The act of consciously maintaining a certain degree of similarity to those in your general social circles  **Social comparisons:** The process by which people evaluate their attitudes, abilities or performance relative to others  **Group norms:** Any behaviour, belief, attitude or emotional reaction held to be correct or acceptable by a given group in society  **Social support:** The apperception or provision of assistance or comfort to others, typically to help them cope with a variety of biological, psychological and social stressors. Support may arise from any interpersonal relationship in an individual’s social network, involving friends, neighbours, religious institutions, colleagues, caregivers of support groups  **Power:** The capacity to influence others, even when they try to resist this influence Intergroup conflict: Disagreement or confrontation between two or more groups and their members. This may involve physical violence, interpersonal discord, or psychological tension.  **Alienation:** Estrangement from one’s social group; a deep-seated sense of dissatisfaction with one’s personal experiences that can be a source of lack of trust in one’s social or physical environment or in oneself; the experience of separation between thoughts and feelings    **Group identity:** the set of behavioural or personal characteristics by which an individual is recognizable [and portrays] as a member of a group | Consider coding to this domain:  Interactions/support with any external partners or organisations that impacted the exercise (or whether they lacked interaction):  e.g., UKHSA/PHE, Labs, GPs, other ODNs, HCT peers, patients and how those interactions influence the conduct of the exercise  Also, how the ODN lead/colleagues interacted with each other and influenced the exercise  Any social pressures they experienced  Group identity (external) – how either the participant or patients are identified as belonging to a particular group  Inappropriate coding to this domain:  Professional-social role/identity related discussions  Environmentally influenced staffing restrictions or availability – code to Environmental context | So the GP then put a stop to us, you know, doing anything there and we just and yeah. We just worked on what they said basically. (ppt 4)  Only you know the… there were few grumpy phone calls with people. You know, ‘why you're contacting me again?’ So, some of the stuff got a, you know, had the occasional unpleasant discussion with somebody, although most of the time that was diffused afterwards (participant 15)  So we have, we had have the field, the epidemiology service on-site umm and which is aligned close with what was back then with Public Health England [now UKHSA] and we essentially collaborated closely with them. (participant 12) |
| 1. **Emotion**   *A complex reaction pattern, involving experiential, behavioural and physiological elements, by which the individual attempts to deal with a personally significant matter or event* | **Fear:** An intense emotion aroused by the detection of imminent threat, involving an immediate alarm reaction that mobilizes the organism by triggering a set of physiological changes  **Anxiety:** A mood state characterized by apprehension and somatic symptoms of tension in which an individual anticipates impending danger, catastrophe or misfortune.    **Affect:** An experience or feeling of emotion, ranging from suffering to elation, from the simplest to the most complex sensations of feelings, and from the most normal to the most pathological emotional reactions.    **Stress:** A state of physiological or psychological response to internal or external stressors  **Depression:** A mental state that presents with depressed mood, loss of interest or pleasure, feelings of guilt or low self-worth, disturbed sleep or appetite, low energy, and poor concentration  **Positive/negative affect:** the internal feeling/state that occurs when a goal has/has not been attained. A source of threat has/has not been avoided, or the individual is/is not satisfied with the present state of affairs Burn-out: Physical, emotional or mental exhaustion, especially in one’s job or career, accompanied by decreased motivation, lowered performance and negative attitudes towards oneself and others | Consider coding to this domain:  Worries/fears/concerns/stress invoked by the exercise  Any exhaustion/burnout  Positive feelings of reassurance  Discussion about times when participant worries more/less  ***Code both positive and negative answers***  Inappropriate coding to this domain:  Optimism/Pessimism or benefits/downsides of the exercise  Feelings about (Long-term) outcomes – Code to Beliefs about Consequences | All this stuff I’d forgotten. Isn’t it funny how you’re bringing it all back? Ohh, that was hideous.  ...I do remember that [doing the letter mailing] was hideous.  (ppt 11)  So it was one of them where you just got on with it, you know, I know something that sit back and moan and groan about it, but I think the energy of the putting to moaning and groaning that could have actually got more done so. mm.  (participant 13) |
| 1. **Behavioural regulation**   *Anything aimed at managing or changing objectively measured actions*    *(capability)* | **Self-monitoring:** A method used in behavioural management in which individuals keep a record of their behaviour, especially in connection with efforts to changes or regulate the self; a personality trait reflecting an ability to modify one’s behaviour in response to a situation  **Breaking habit:** to discontinue a behaviour or sequence of behaviours that is automatically activated by relevant situational cues  **Action planning:** The action or process of forming a plan regarding a thing to be done or a deed. | Consider coding to this domain:  Their involvement in re-engagement strategy/planning meetings  Procedures/monitoring that ODN already uses or started using in the exercise to track performance  Troubleshooting  Record keeping  Clear examples of action planning, self-monitoring, feedback processes (e.g. on their performance)  Things participant does to establish a routine  Strategies used to overcome challenges, and manage the exercise targets over time  Strategies/meetings to manage future re-engagement afterwards due to the exercise (prefix “post”)  Inappropriate coding to this domain:  Outline descriptions of how they did the exercise (code outside of TDF to ‘approach’)  Resources they had in place that helped them manage – code to Environmental Context and resources  Consequences for their ODNs performance due to the exercise – code to beliefs about consequences. | I remember...setting an hour aside one day and seeing how many patients I could scrutinize because I also had to register them on our hospital system so that I could do the letters. ...So I remember spending an hour one day seeing how many I could scrutinize in an hour and how many I could register on our hospital system in an hour.  ... Then I worked out how many in total I had to do and how many hours I needed. ...  So that was where this setting protected time an hour every day for upteen weeks to do that part of the job. (ppt 11)  So it's quite a protracted, was quite protracted process. So the data was shared from PHE at the time with our clinical lead. We then had a- the ODN had a series of meetings with various trust executives and information governance staff about handling this data.  ...  And so we had lots of discussions around how what was the best way to do this? How would we contact people …we decided that first of all we would break down the list of names into our spoke sites, our geographical areas that we would send the information out to each of our spoke hospital sites for them to see if the names were known to them at all in their HCV service historically or currently… (ppt 11) |

**Additional coding outside the TDF**

| **Method of conducting the exercise** | Neutral descriptions of what they did, in line with the guidance to the exercise.  Be careful to distinguish from behavioural regulation (involving some sort of monitoring or planning process)  Be careful to distinguish from how these behaviours influenced the outcomes/exercise (ie barriers and facilitators) | How we conducted it, we received a list of patients uh, a full list from umm… I keep forgetting to call it public, but at the time it was Public Health England.... And we put together the demographic information and what we did with that demographic information, we then looked to see which of those was still within our operational delivery network and which had moved away. (ppt 14) |
| --- | --- | --- |
| **Recommendations** | Any recommendation made by participant for future re-engagement or other HCV elimination work  Their beliefs about how to tackle re-engagement for the remaining cohort/elimination targets and descriptions of different re-engagement activities that do not resemble this exercise | hopefully if they do send it out again then it will be up to date with addresses and everything else because that’s obviously an issue (ppt 4).  It's for somebody you know above my pay grade to decide what they would, how they would approach that [engaging the last cohort of Hep C people] because you know weren't necessarily approach that at the same, the same way would you. (ppt 4) |
