# Supplementary material for: Qualitative evaluation of the barriers and facilitators to a retrospective hepatitis C virus patient re-engagement exercise in England
Source: BMJ Open. 2025 Nov 13;15(11):e104546. doi: 10.1136/bmjopen-2025-104546 (PMC12625882; doi:10.1136/bmjopen-2025-104546)
Supplement: online supplemental file 3 [file bmjopen-15-11-s003.docx]

**Supplementary materials – Tables of themes**

Supp. Table 1. Full list of themes of barriers and facilitators, in alphabetically order by theoretical domain from the TDF, with number of participants and example quotes. Mixed themes are both barriers and facilitators.

| Theoretical domain – Theme name | Number of participants | Barrier/ Mixed/ Facilitator | Example quote |
| --- | --- | --- | --- |
| Behavioural regulation: *Anything aimed at managing or changing objectively measured actions* | | | |
| Held regular strategy meetings | 5 | Facilitator | “we had lots of discussions around how what was the best way to do this? How would we contact people…” (Participant 11) |
| Adopted methodical approach to the exercise | 6 | Facilitator | “what we did is, we gave ourselves targets of right, we're doing so many per day because it were such a big task, so we'd block the diary out at least once a week to do a big chunk of that data. So that was the kind of process that we kind of followed.” (Participant 21) |
| Beliefs about capabilities: *Acceptance of the truth, reality, or validity about an ability, talent or facility that a person can put to constructive use* | | | |
| Confidence doing the exercise | 9 | Mixed | “I felt very confident in what I was doing because I had a good line manager, and I had a good doctor that was leading the project.” (Participant 20) |
| Beliefs about consequences: *Acceptance of the truth, reality or validity about outcomes of the exercise* | | | |
| Treatment yield and other outcomes from the exercise | 19 | Mixed | “we have actually had quite a good response in terms of the number of people we've been able to start on treatment” (Participant 1). |
| Worried about people’s responses to the exercise | 8 | Mixed | “The only thing that sort of we were a bit worried about in the beginning was the potential implications of someone kicking off getting a letter that they perhaps was incorrect. So that was a bit concerning for us” (Participant 23) |
| The exercise enabled more organised ways of reengaging patients | 7 | Facilitator | “I think what [the re-engagement exercise has] done is it’s kind of spurred on this whole sort of find-and-treat, re-engagement, [buy-in] plan and going and finding these guys.” (Participant 0) |
| Due to success, continued to conduct the re-engagement exercise (in some form) | 6 | Facilitator | “we also have now an ongoing relationship with [UKHSA]… regularly provides us lists of people …So, that is just a free-flowing conversation now. So, almost reengagement is part of our work stream” (Participant 14) |
| Emotion: *Complex reactions by which the individual attempts to deal with the feelings invoked by the exercise* | | | |
| Reappraised negative feelings into positive feelings/behaviours | 5 | Mixed | “it was one of them where you just got on with it, you know, I know some that sit back and moan and groan about it, but I think the energy of the putting to moaning and groaning that could have actually got more done so. mm.” (Participant 13) |
| Stress and other negative emotions | 12 | Barrier | “unfortunately when you get a spreadsheet with that amount of data on it is. It's just overwhelming, really, to be honest with you.” (Participant 16) |
| Environmental context and resources: *Any circumstance of a person’s situation or environment that discouraged or encouraged them to work on the exercise* | | | |
| ODN and NHS system resource restrictions | 6 | Barrier | “across the ODN we couldn't have asked each spoke to do it. They would not have been able to send out the letters…They didn't have the resource, the staffing or the functionality that we've got with our electronic patient record. And I, as I understand it as well, the data when it was released was the responsibility of the clinical lead.” (Participant 11) |
| Limited testing or treatment facilities | 5 | Barrier | “when the reengagement exercise happened, we were just in that scale up phase of treatment…there was a balancing act because we had a lot of people waiting… when it was just cirrhotics or severe liver disease only. So, actually, timing of finding people was quite important because what we didn't want to do was find people and then say, ‘oh, and by the way, you'll have to wait another year for your treatment.’” (Participant 15) |
| Low staff and staff turnover caused disruption | 16 | Barrier | “as was probably the case with everyone, there wasn't a dedicated team just for that programme. And so, it was sort of done at the end of… well it was done alongside lots of other things” (Participant 25) |
| Referral processes, data access and governance issues | 7 | Barrier | “I didn't actually work out of [the other] hospitals, but I contacted them from [my hospital] so it was quite a large thing to set up because you know …. setting up an honorary contract actually had to go and do courses in order to use their systems.” (Participant 11) |
| *Laboratories*: Some existing access, but no capacity to gain access to all laboratories | 14 | Mixed | “I mean, we already had good links with our labs because we were setting up alerts, so we could kind of identify and get reports from them and that kind of was quite helpful as part of the data cleansing initially” (Participant 1) |
| COVID19 affected the exercise | 15 | Mixed | “We still carried on treating and testing patients for hepatitis C, but this exercise really went on hold and we came back to it after COVID essentially.” (Participant 12) |
| There were useful ODN/Trust resources, databases, and systems | 10 | Facilitator | “…we had quite good systems in place for managing hep c treatment and understanding who had already gone through that” (Participant 1) |
| Goals: *Mental representations of outcomes that individuals want to achieve that relate to the exercise* | | | |
| Prioritised people with PCR RNA test results | 4 | Mixed | “We did try and contact them [patients with only antibody results] and but they definitely sat probably further down our list of priorities for contacting” (Participant 9) |
| We had no fixed targets, which enabled accuracy and flexibility | 6 | Mixed | “there were no targets, no strict deadlines, it was to be done in the safest possible way. So that's how we operated. We would write them in the safest possible way. We wouldn't rush it for the sake of a couple of weeks, it was all about accuracy.” (Participant 14) |
| The exercise fitted well with targets and/or elimination goals | 9 | Facilitator | “[the re-engagement exercise] was one of the key things that we were doing 'cause- I mean my view was well, we know that there's several probably thousand people that we know have got hep C versus we can go and test. And it just seemed an obvious place to go. So it was a priority for us.” (Participant 23) |
| Intention: *A conscious decision to perform a behaviour or a resolve to act in a certain way* | | | |
| The exercise was necessary but varied beliefs on whether to do it again | 6 | Mixed | “I do think it was valuable. Whether I'd want to do it again in the same way, I'm not sure.” (Participant 22) |
| Commitment to the exercise | 4 | Facilitator | “we were committed. It was committed to that letter and doing it this way. Yeah.” (Participant 11) |
| Knowledge: *An awareness of the existence of the components of the exercise/how to do the exercise* | | | |
| The data contained discrepancies or errors | 4 | Barrier | “I think actually a lot of things that were sent to us were people that had self-cleared and it was proven or we got false positives, things like that or actually people were sent through as being positive, but they weren't positive” (Participant 17) |
| There was a lack of up-to-date data about patients | 11 | Barrier | “with a lot of people, we could no longer find their… checking the NHS Spine...can't remember if it gave us addresses on the spreadsheet, but anyway we updated via the Spine to get more contemporaneous contact details with varying success” (Participant 25) |
| Memory attention decision making processes: *The ability to retain information, focus selectively on aspects of the exercise and choose between two or more alternatives* | | | |
| The exercise required a lot of time and attention | 16 | Barrier | “So, it was quite obviously intense because you were writing to these patients, you were getting blood results back and forth. So, it was about two and a half to three days a week of my time” (Participant 14) |
| It was hard to attend to amongst other priorities | 10 | Barrier | **“**It was always in the diary as a priority, but as, as I'm sure with yourself, if something comes in that's more pressing, it does have to. It did have to go on the back burner.” (Participant 21) |
| Optimism: *The confidence that things will happen for the best or that desired goals will be attained* | | | |
| Pessimistic or unsure that the exercise would help re-engage patients | 9 | Barrier | “I would err towards pessimistic because...We’ve done similar things to patients, so we write to patients who have been referred to us who we know have got hepatitis C who are RNA positive, who don’t engage with us....So I knew that that we probably weren’t going to get much from this.” (Participant 11) |
| Optimistic/became optimistic that the exercise would help re-engage patients | 9 | Facilitator | “Yeah, I mean, but these are a hard to re-engage groups, you know, by definition. But very optimistic that there was now a process in place to at least try” (Participant 0) |
| Reinforcement: *Increasing the probability of a response by arranging a dependent relationship, or contingency, between the incentives/benefits/rewards/punishments and the exercise* | | | |
| Received punishment due to errors or complaints | 3 | Barrier | “obviously we apologised about [sending some letters in error] and I think [clinical lead] had to speak to the [patients] on that and I obviously got a wrap on the knuckles because I should have checked” (Participant 4) |
| The exercise was personally rewarding | 10 | Facilitator | “I did [find it personally rewarding]. Because you get people treated, people that didn’t know they had the virus” (Participant 4) |
| Skills: *An ability or proficiency in conducting the exercise* | | | |
| IT/data skills/screening training were needed | 8 | Barrier | “if you've got an IT geeky person that and I'm not, that knows how to do cross referencing of databases they could probably do things in a push of a button, as we're all just laboriously going through individual names case by case.” (Participant 16) |
| The exercise guidance was flexible and adaptable, which had advantages and disadvantages | 8 | Mixed | “I guess we got a high-level guidance essentially, but it was you know it was up to us to work out how we you know to work out the detail essentially.” (Participant 12) |
| Social influences: *Interpersonal processes that can cause individuals to change their thoughts, feelings, or behaviours during their conduct of the exercise* | | | |
| *UKHSA/NHSE^[[1]](#footnote-2)^*: Perceived pressure to conduct the exercise | 10 | Mixed | “I think all ODNs had pressure to do the exercise because it was mandated by NHS England. So yeah, there was pressure to do it and we obviously wanted to do it in the best way that we could as well.” (Participant 16) |
| *UKHSA/NHSE*: Interactions with UKHSA/NHSE during the exercise | 17 | Mixed | “When NHS England put out a project they don’t never have a project plan, you know and you know they try their hardest to do what they need to do in the time they need to do it. But it’s not always a very clear message” (Participant 6) |
| *Within ODN:* Colleagues in our ODN were collaborative | 10 | Facilitator | “our ODN is collaborative and there are good communication channels.” (Participant 11) |
| Links with other ODNs | 4 | Mixed | “So, for example, with [other ODN] we already were in semi-regular communication with the ODN clinical lead [name] up there. So I suppose that kind of helped when transferring patients across. “ (Participant 1) |
| *GPs:* Cooperation/support, or lack thereof, from GPs | 19 | Mixed | “that’s really where it fell down because the GP practises didn’t have the time to sift through the amounts of stuff that we were sending them. So we were really at a bit of an impasse as to know whether it was appropriate to contact that person. So we left it and then we did the whole exercise again and we did get a few back. Few GPs did respond.” (Participant 6) |
| *Patients*: It was difficult to make contact with people/patients | 12 | Barrier | “I would say at least half the attempts to contact the people probably never got to the individual, which is a big factor in all of this” (Participant 23) |
| *Patients*: Most people/patients happy to be contacted, but a small number of difficult conversations were had | 15 | Mixed | “One [patient phoned] to say “I’ve been treated in a trial. Why don’t you know that you shouldn’t have contacted me.” And the second one to say “this was delivered to me on a Friday and I couldn’t get through on the weekend and then the week after the [clinical nurse specialist] mobile wasn’t answered and I had a week of extreme anxiety” (Participant 25) |
| *Patients*: Some patients don’t want treatment now | 6 | Barrier | “It’s just there is like a few people that are quite elderly. And I think it’s probably a mixture of embarrassment, a mixture of knowing what the previous treatments were like, the fear. And the embarrassment and also the effect that it may have on them financially that they just can’t. Can’t let that go, you know.” (Participant 6) |
| Social/professional role and identity: *Behaviours or qualities of the individual or organisation in their work setting, and how these influenced the exercise* | | | |
| Appropriateness of ODN conducting the re-engagement exercise | 17 | Mixed | “[the Hep C elimination programme is] being done by the wrong people in that we haven't. It's being led by ODN, Operational Delivery Networks across the country and led by clinicians who haven't had any training in public health or screening or surveillance.” (Participant 12) |
| Appropriateness of individual roles for tasks | 16 | Mixed | “So we were very clear of who's clinical and who's nonclinical. So by us owning this, we knew what we had to do, where the nurses could get on and do whatever else they wanted.” (Participant 13) |
| The ODN ethos is that you should not give up on people | 6 | Facilitator | “Some of the provider trusts would stringently apply their fail to attend policy. So one fail to attend, discharge. We've completely changed that. We don't discharge anybody. We give them, you know, if somebody says I'm not interested, I'm not in a good place. OK, we'll contact you again in three months.” (Participant 22) |
| Specialist nurses were crucial to the exercise | 6 | Facilitator | “I think also utilising the local nurses own knowledge as well. Yeah my nursing team, [who have been in post for many years], they know the patients quite regularly their knowledge, for them is phenomenal really having them look through the patient groups. Was quite important for us” (Participant 8) |

Supp. Table 2. Themes of barriers encountered before or part way through the exercise, resulting in adaptations that then facilitated the exercise. Included here are the more frequent themes.

| Relevant barrier encountered or anticipated (from Supp. Table 1) | Adaptations that became facilitators | Number of participants | Example quote |
| --- | --- | --- | --- |
| Beliefs about consequences: worried about patients responses to the exercise | Provided patient support | 4 | “what we agreed was anyone who was distressed could come into a… walk into a [day] clinic and just be tested then and there. So, or they were offered some reassurance. So that's how we did it. We pretty much managed it, you know, really well at that point.” (Participant 14) |
|  | Getting the language in letters right/providing minimal information | 6 | “…although we were checking on the Spine to sort of see where patients lived, if that was their current address, you never know if it ended up in the wrong place. So the nurses would never put what the diagnosis was in the letter that I just said. You've had a historic blood test and we just need to talk to you.” (Participant 13) |
| Social influences - *Patients*: It was difficult to make contact with people/patients | Included GPs more in communications | 7 | “[after trying to contact patients] we'll write to the GP and make them aware that we've tried everything we can. They're not engaged. But if they want to be re-referred to the service we will happily offer them the next available appointment. I have had cases where once that letter was sent [to the GP], the patient received a copy of that letter and it actually spurred them to engage.” (Participant 24) |
| Social influences - *Patients*: It was difficult to make contact with people/patients;  Patients do not want treatment now | Offered local testing so people didn’t have to travel | 4 | “They [patients] love the fact that we go to them. And. They pick where we meet them. It amazed me at first. How many people let you park outside their house” (Participant 22) |
| Social influences *- Patients*: It was difficult to make contact with people/patients | Timed contact to when patient support / treatment was available | 3 | “We modified our protocols, so we never sent out any letters again on a Friday or around the bank holiday, only on Monday.” (Participant 25) |
| Social influences - *Patients*: It was difficult to make contact with people/patients; Patients do not want treatment now | Used multi-pronged engagement processes | 7 | “We decided to take a slightly different approach to it because we didn't think we were really gonna get the desired effect. I suppose by just sending a letter to both GPs and or patients, so we thought it would be better to kind of do a more hands on approach I think, as well as sending the letters, of course, but we want it to be a bit more. You know, I suppose going a bit deeper in terms of the communication we were gonna try.” (Participant 1) |

1. Participants occasionally misattributed the origin of the data and exercise, hence we combined UKHSA/NHSE under one social influence. [↑](#footnote-ref-2)
