# Supplementary material for: Qualitative evaluation of the barriers and facilitators to a retrospective hepatitis C virus patient re-engagement exercise in England
Source: BMJ Open. 2025 Nov 13;15(11):e104546. doi: 10.1136/bmjopen-2025-104546 (PMC12625882; doi:10.1136/bmjopen-2025-104546)
Supplement: Supplementary file 4 [file bmjopen-2025-104546.reviewer_comments.pdf]

PEER REVIEW HISTORY

BMJ Open publishes all reviews undertaken for accepted manuscripts. Reviewers are asked to complete a checklist review form and are provided with free text boxes to elaborate on their assessment. These free text comments are reproduced below.

ARTICLE DETAILS

Title (Provisional)

Qualitative evaluation of the barriers and facilitators to a retrospective Hepatitis C Virus patient re-engagement exercise in England

Authors

Stuart, Avelie; Hoerst, Carina; Etoori, David; Lorencatto, Fabiana; Rosenberg, William; Lowndes, Catherine; Simmons, Ruth; Mandal, Sema; Gillyon-Powell, Mark; Desai, Monica

---

VERSION 1 - REVIEW

**Reviewer**	**1**
**Name**	**Senoo-Dogbey, Vivian Efua**
**Affiliation**	**University of Ghana, PUBLIC HEALTH**
**Date**	**29-Jun-2025**
**COI**	**None**

Title and Abstract
The study has a clear and descriptive title accurately reflects the study's scope and design. The abstract is structured well with background, objective, methods, results, and conclusions clearly outlined.

1. However, consider tightening the abstract by highlighting unique contributions more explicitly.
2. Adding more concise policy implications could strengthen impact.

Introduction
The background provides clear epidemiological and policy context for HCV re-engagement. and justifies the qualitative evaluation using the TDF framework.

1. The study could better integrate international comparisons to demonstrate novelty.
2. The study should consider clarifying the rationale for focusing solely on ODN staff perspectives.

Methods
The study described a well-designed sampling strategy covering regional variation and treatment yield strata. Additionally it gives a clear application of TDF with combined inductive and deductive analysis.

1. The study could benefit from clearer explanation of how data saturation was assessed and declared.
2. The study needs to include details on how inter-coder reliability was ensured.

Results
The study comprehensively reported barriers, facilitators, and adaptations and used extensive direct quotes to support findings.

1. Tables are detailed but could be streamlined for easier reader navigation.
2. Consider highlighting more unexpected findings or contradictions within themes.

Discussion

This section is well-linked to literature and situates findings within UK and global elimination targets. Practical recommendations are clear and directly informed by participants' views

1. Further reflection on generalizability beyond England would strengthen the paper.
2. Could you discuss how these insights can inform other infectious disease re-engagement efforts.

Limitations
The study is transparent about recall bias and limited PPI. It acknowledges COVID-19 impact and variable GP involvement.

1. There is the need to discuss limitations of not including patient perspectives directly in this paper.

Recommendations and Policy Implications

2. Practical, actionable recommendations for improving future exercises.
3. Clear mapping to behavioural intervention functions adds rigour.
4. The authors could add more emphasis on resource allocation and sustainable funding for follow-up.

Conclusion
The study concludes with clear takeaways for national strategy and ODN practice.

1. A more forceful statement about the critical role of continued qualitative evaluation could be included.

References

References are up-to-date and relevant to HCV elimination and qualitative methods. Double-check consistency of citation format.

Overall Recommendation
This manuscript presents robust qualitative insights with practical implications for re-engaging HCV patients.

It is well-written and methodologically sound. The findings will be useful for policymakers and practitioners.

**Reviewer**	**2**
**Name**	**Higgs, Peter**
**Affiliation**	**Burnet Institute, Behaviours and Health Risks**
**Date**	**25-Jul-2025**
**COI**	**none**

Thanks for the opportunity to provide some feedback on this interesting and important qualitative manuscript on the ambitious project to retrospectively engage people for hepatitis C treatment and care. The UK health system may well lend itself to be in a better position that many other countries in being able to use retrospective surveillance data - positioning how the UK health system works may be useful for readers unfamiliar with the way it is organised. There are a number of important insights from the data that are likely to be of interest and utility to others embarking on such a project.

Overall the paper uses a descriptive approach to report their data using the TDF to identify challenges to the implementation of this project. This approach is fine but it is not clear or justified why all the domains from the TDF are included - as it reads there are just point by point responses to each of the domains of the TDF. In this way the manuscript reads as a description of the whole process involved in the completion of this project and more like a report to the funding agency than it does as a manuscript. The report for government from Jan 2025 'Evaluation of the hepatitis C: patient re-engagement exercise' is also available online: https://www.gov.uk/government/publications/evaluation-of-the-hepatitis-c-patient-re-engagement-exercise/evaluation-of-the-hepatitis-c-patient-re-engagement-exercise

Noting the point raised above, as a qualitative evaluation I found the use of tables and 'counting' responses from participants took away from the insights provided in the participant narratives collected over the course of the study. There are clearly some important learnings from the completion of this project that will be of advantage to others interested in undertaking such a task - either with hepatitis C or another similar surveillance activity. How can the authors get the most from these participant insights and present them in a way that is more focused on the key lessons learned.

This may be a personal preference for the way qualitative data are presented but I wondered whether the authors could go back and consider which findings are best presented to tell their story of how this exercise developed for those involved in completing it. It may be that

the main findings as presented in their report to government are one way to do this. Or another is to think about the paper being focused on 'lessons learned'.

This means some re-organising of the results (possibly using the same headings and sub-headings) but including the narratives as part of the manuscript rather than in tables. If the focus of the manuscript becomes 'key learnings' then perhaps this would make the paper easier to read and tell the story of the findings in a way that is more readable for people.

Some other minor suggestions below for the authors to consider in any re-working of their manuscript.

The opening sentence (lines 55-57) could include estimates on the actual number of people living with chronic hepatitis C rather than just stating it has substantially fallen esp given there is a proportion of 78.3% - hard to know what this proportion represents without an estimate. The 55,000 people who are estimated as lost to follow up is hard to contextualise without an overall number of people who have been diagnosed with hepatitis C.

On line 107 - the phrase 'response rates from local residents' is used but not entirely clear what that means ? is this local as opposed to new migrants or what exactly?

Data Analysis

It is not clear here whether only 3 transcripts were used in the 'development of the codebook...' and whether these 3 transcripts were also included to 'inductively identify themes...'

I appreciate the transparency of providing the full codebook but wonder whether it is enough to include this as supplementary materials for the manuscript.

VERSION 1 - AUTHOR RESPONSE

Reviewer: 1

Dr. Vivian Efua Senoo-Dogbey, University of Ghana

Reviewer comments	Authors response
**Title and Abstract**<br>The study has a clear and descriptive title accurately reflects the study's scope and design. The abstract is structured well with background, objective, methods, results, and conclusions clearly outlined.<br>1. However, consider tightening the abstract by highlighting unique contributions more explicitly.	Thank you for the positive comments.<br><br>1. We have rephrased the results in the abstract, to focus more on the meaning of the findings and the contributions of the evaluation.<br><br>"Interviewees reported the most barriers in environmental context and resources (including staffing limitations, interruptions during COVID19, restricted laboratory

2. Adding more concise policy implications could strengthen impact.	access), and social influences (with limited responses from GPs and patients). Interviewees discussed whether it was appropriate for ODNs and individual staff to be assigned the data validation work and reported some stress and memory/attention barriers due to the volume of the exercise. They had varied beliefs about the consequences of the exercise, with most believing it was worthwhile due to treatment yield, lessons learned, and confirmation that some people had cleared the virus. Further facilitators included the ODN goals fitting with the exercise, and regional resources such as patient databases. Interviewees also reported adaptations to the exercise that facilitated patient contact, and their ongoing work to re-engage patients emphasised outreach partnerships and peer support."<br><br>2. We also extended the point about policy implications to say, "Government support and funding-provision for regionally tailored holistic re-engagement approaches, alongside enhancements to health surveillance data, could enable barriers to re-engagement work to be overcome."
**Introduction**<br>The background provides clear epidemiological and policy context for HCV reengagement and justifies the qualitative evaluation using the TDF framework.<br>1. The study could better integrate international comparisons to demonstrate novelty.<br>2. The study should consider clarifying the rationale for focusing solely on ODN staff perspectives.	1. We have observed few international comparisons in the literature. The exercise in the Netherlands is the closest international parallel we've found. We have also added references to a similar reengagement exercise conducted in Wales (UK), and a sentence to point out that "Qualitative evaluations of any of reengagement exercises are lacking in the published literature." (p. 4, ln 107).<br><br>2. This is a good point. We added a sentence to the introduction (p. 5, ln 119):<br>"Due to the amount of time that had passed, we were unable to involve patients in the evaluation."
**Methods**<br>The study described a well-designed sampling strategy covering regional variation and treatment yield strata. Additionally it gives a clear application of TDF with combined inductive and deductive analysis.	Thank you again.<br>1. We added a point about our decision for stopping data collection, (p. 7, ln 158):<br><br>"Rather than data saturation, our decision to end data collection occurred when our recruitment strategy was exhausted, as some staff working on the exercise were no longer

1. The study could benefit from clearer explanation of how data saturation was assessed and declared.<br>2. The study needs to include details on how inter-coder reliability was ensured.	employed in the ODN and we had exceeded our estimated sample size. We also observed a high frequency of common themes across the interviews (see Results)."<br><br>2. The type of thematic analysis we used does not involve calculating inter-coder reliability, due to the analysis being a reflexive process involving the researcher interpretation of the data. However, the team reviewed the coding to ensure the TDF framework was being applied consistently and rigorously (see p.7 ln 169)
**Results**<br>The study comprehensively reported barriers, facilitators, and adaptations and used extensive direct quotes to support findings.<br>1. Tables are detailed but could be streamlined for easier reader navigation.<br>2. Consider highlighting more unexpected findings or contradictions within themes.	1. We have streamlined the main table (p. 9), also in response to Reviewer 2's feedback about moving quotes into the narrative.<br><br>2. We edited some the theme descriptions throughout the results to highlight more unexpected/contradictory findings.<br><br>For example, on p. 12, ln 269, "Unexpectedly, a few interviewees said the pandemic freed up time that allowed them to focus on the exercise."<br><br>p. 13, Ln 303:<br>"However, other interviewees saw the exercise as reflecting their role and thus they were able to prioritise it."<br><br>p. 14, Ln 325:<br>"Unexpectedly, many interviewees did not feel that any additional training would have been beneficial due to having ODN-specific systems and processes."
**Discussion**<br>This section is well-linked to literature and situates findings within UK and global elimination targets. Practical recommendations are clear and directly informed by participants' views<br>1. Further reflection on generalizability beyond England would strengthen the paper.<br>2. Could you discuss how these insights can inform other infectious disease reengagement efforts.	We have addressed both of these points in the following paragraph in the discussion (p. 24, ln 554)<br><br>"WHO Europe published the NHSE-UKHSA re-engagement exercise as an example of best practice (5) and this qualitative evaluation contributes insights into building on the re-engagement exercise successes should it be considered again, or attempted in another setting or country. Interestingly, the delivery of this exercise was structured according to the NHS health services in

	England and yet achieved a similar yield to the nationally rolled out retrieval exercise in the Netherlands (11), and a similar exercise by NHS Wales (15). In the Netherlands they reported greater success their smaller regional trial (12), and in this exercise ODNs also reported regional and environmental influences (e.g. access to laboratories, staff availability, the varied impact of COVID19, and local adaptations to patient contact and support). Regional resources and processes may explain the variation in return of data to UKHSA and the exercise outcomes (6). Key lessons to be learned, if applying this approach to other settings or infectious diseases, would be the need for regionally commissioned and tailored approaches with sufficient resource and training to support data validation, and the use of outreach or patient-centred care methods to supplement any patient letter mailing approaches."<br><br>The paragraph that follows (ln 569) also discusses international qualitative studies on hepatitis C management strategies (although not re-engagement exercises).
**Limitations**<br>The study is transparent about recall bias and limited PPI. It acknowledges COVID-19 impact and variable GP involvement.<br>1. There is the need to discuss limitations of not including patient perspectives directly in this paper.	We have added a statement about why patient perspectives were not included in this study, which was because of the amount of time that had passed since the exercise began (2018-19), we would not have been able to adequately sample patients (p. 26, ln 594)<br><br>"we did not include patient perspectives in this study, as the amount of time passed would limit our ability to adequately recruit patients who were contacted during the re-engagement exercise. Because it is important to understand patient perspectives, we are also conducting a stand-alone patient interview study about factors affecting their re-engagement in care after a period of disengagement."
**Recommendations and Policy Implications**<br>2. Practical, actionable recommendations for improving future exercises.<br>3. Clear mapping to behavioural intervention functions adds rigour.	Thank you for this important point. We have added this sentence "To enable all of these improvements to take place, funding and dedicated resources are vital to ensuring the sustainability of re-engagement initiatives, including joint efforts with other disease elimination strategies (29)." (p. 27, ln 620)

4. The authors could add more emphasis on resource allocation and sustainable funding for follow-up.	This includes a link to a new report on sustaining success in elimination initiatives.
**Conclusion**<br>The study concludes with clear takeaways for national strategy and ODN practice.<br>1. A more forceful statement about the critical role of continued qualitative evaluation could be included.	Thank you. We have added the sentence "Very few such initiatives have been attempted globally, and qualitative evaluation is vital to investigating the barriers, facilitators, and lessons that can be learned." (p. 27, ln 626).
**References**<br>References are up-to-date and relevant to HCV elimination and qualitative methods. Double-check consistency of citation format.	We have checked and made corrections to the citation format.
**Overall Recommendation**<br>This manuscript presents robust qualitative insights with practical implications for reengaging HCV patients.<br>It is well-written and methodologically sound. The findings will be useful for policymakers and practitioners.	Thank you for the positive feedback on our manuscript.

Reviewer: 2
Dr. Peter Higgs, Burnet Institute, La Trobe University, La Trobe University
Comments to the Authors:

**Reviewers comments**	**Authors response**
Thanks for the opportunity to provide some feedback on this interesting and important qualitative manuscript on the ambitious project to retrospectively engage people for hepatitis C treatment and care. The UK health system may well lend itself to be in a better position that many other countries in being able to use retrospective surveillance data - positioning how the UK health system works may be useful for readers unfamiliar with the way it is organised.<br><br>There are a number of important insights from the data that are likely to be of interest and utility to others embarking on such a project.	We have added a bit more context on the UK health system, as follows (p. 3, ln 57): "Hepatitis C Operational Delivery Networks (ODNs) are secondary care providers that were established by NHSE in 2015 and charged with managing HCV treatment, providing impartial clinical advice and expertise, and coordinating patient pathways across different services and providers, within a geographical catchment area, to ensure equitable and high standards of care. People can be referred to ODNs via primary care, drug and alcohol services, and sexual health/genitourinary clinics. The UKHSA is an arms-length government body that conducts hepatitis C surveillance using laboratory records, to support public health responses to infectious diseases"

Overall the paper uses a descriptive approach to report their data using the TDF to identify challenges to the implementation of this project. This approach is fine but it is not clear or justified why all the domains from the TDF are included - as it reads there are just point by point responses to each of the domains of the TDF. In this way the manuscript reads as a description of the whole process involved in the completion of this project and more like a report to the funding agency than it does as a manuscript.<br>Noting the point raised above, as a qualitative evaluation I found the use of tables and 'counting' responses from participants took away from the insights provided in the participant narratives collected over the course of the study. There are clearly some important learnings from the completion of this project that will be of advantage to others interested in undertaking such a task - either with hepatitis C or another similar surveillance activity. How can the authors get the most from these participant insights and present them in a way that is more focused on the key lessons learned.<br>This may be a personal preference for the way qualitative data are presented but I wondered whether the authors could go back and consider which findings are best presented to tell their story of how this exercise developed for those involved in completing it. It may be that the main findings as presented in their report to government are one way to do this. Or another is to think about the paper being focused on 'lessons learned'.<br>This means some re-organising of the results (possibly using the same headings and sub-headings) but including the narratives as part of the manuscript rather than in tables. If the focus of the manuscript becomes 'key learnings' then perhaps this would make the paper easier to read and tell the story of the findings in a way that is more readable for people.	Thank you for these comments. On reflection, the themes did indeed read as too point-by-point. We have changed several things to improve the way the findings are reported.<br><br>First, we have re-ordered the themes in progression of how the exercise developed, and extended the narrative of how barriers/facilitators arose during the exercise. We also make more links between themes/TDF domains, and incorporated the previously separate 'adaptations to the exercise' themes within the main results section to highlight how participants learnt to improve the exercise as they developed it (p. 19, ln 451).<br><br>Second, we moved quotes from the tables and placed them in the appropriate place in the narrative, which we believe does improve readability and makes better use of the quotes to illuminate the themes.<br><br>Third, as suggested we removed the counts of participants from the narrative - although not from the table, as we thought some people may want this information. We agree this puts the emphasis more on the richness of the qualitative data rather than frequencies.<br><br>Fourth, we moved a few themes to the full list of themes in the supplementary materials. These themes were more descriptive/list-like and did not contribute to the lessons learned from the exercise. We believe this makes it easier for the reader to focus on what was learnt from the exercise.<br><br>Please note that because of the extensive restructure we did not highlight each change in the document.<br><br>Unlike the main themes, we kept the recommendations section quotes within the tables (p. 22, tables 4 & 5), as we felt this section serves as a summary of additional insightful reflection from participants, rather

	than pertaining to directly to the evaluation of the exercise. However, we are happy to reconsider if the reviewers feel it would be better in a different format.
The opening sentence (lines 55-57) could include estimates on the actual number of people living with chronic hepatitis C rather than just stating it has substantially fallen esp given there is a proportion of 78.3% - hard to know what this proportion represents without an estimate. The 55,000 people who are estimated as lost to follow up is hard to contextualise without an overall number of people who have been diagnosed with hepatitis C.	We added in the numbers to provide context (p. 2, ln 47)<br><br>"The number of people living with chronic hepatitis C virus (HCV) in England has fallen 30% since 2015 to an estimated 89,900 people in 2020 (1). An estimated 55,000 of those people had been lost to follow up at the time (2)"
On line 107 - the phrase 'response rates from local residents' is used but not entirely clear what that means ? is this local as opposed to new migrants or what exactly?	We have clarified that the comparison was to people living outside of the province (p. 4, ln 103)
Data Analysis<br>It is not clear here whether only 3 transcripts were used in the 'development of the codebook...' and whether these 3 transcripts were also included to 'inductively identify themes...'<br><br>I appreciate the transparency of providing the full codebook but wonder whether it is enough to include this as supplementary materials for the manuscript.	The explanation of the data analysis has been rephrased to clarify that 3 transcripts were used for the deductive codebook development, and all transcripts used in the inductive thematic analysis (p. 7, ln 168)<br>"...development of codebook using three transcripts coded by the first author to aid the deductive analysis to the TDF domains, 3) discussing and revising the codebook with the team (see supplementary materials for final version), 4) inductively identify themes within domains using all of the transcripts..."<br><br>The codebook is only intended to be included in the supplementary material.

VERSION 2 - REVIEW

Reviewer 2

Name Higgs, Peter

Affiliation Burnet Institute, Behaviours and Health Risks

Date 28-Sep-2025

COI

Thank you for taking the time to comprehensively respond to the range of comments from both reviewers. The responses show that you have taken on board the issues raised and you

have worked to ensure that this has improved the readability of the manuscript. Authors have also included materials as appendix appropriately.
